# CPEB3 regulates neuron-specific alternative splicing and involves neurogenesis gene expression

**DOI:** 10.18632/aging.202259

**Published:** 2020-12-09

**Authors:** Wenrui Qu, Hongjuan Jin, Bing-Peng Chen, Jun Liu, Rui Li, Wenlai Guo, Heng Tian

**Affiliations:** 1 Department of Hand Surgery, The Second Hospital of Jilin University, Changchun, Jilin Province, China; 2 Department of Plastic and Reconstructive Surgery, The First Hospital of Jilin University, Changchun, China; 3 Orthopedic Medical Center, The Second Hospital of Jilin University, Changchun, Jilin Province, China

**Keywords:** neuron, RNA-binding protein, CPEB3, alternative splicing, neurogenesis

## Abstract

In the mammalian brain, alternative pre-mRNA splicing is a fundamental mechanism that modifies neuronal function dynamically where secretion of different splice variants regulates neurogenesis, development, pathfinding, maintenance, migration, and synaptogenesis. Sequence-specific RNA-Binding Protein CPEB3 has distinctive isoform-distinct biochemical interactions and neuronal development assembly roles. Nonetheless, the mechanisms moderating splice isoform options remain unclear. To establish the modulatory trend of CPEB3, we cloned and excessively expressed CPEB3 in HT22 cells. We used RNA-seq to analyze CPEB3-regulated alternative splicing on control and CPEB3-overexpressing cells. Consequently, we used iRIP-seq to identify CPEB-binding targets. We additionally validated CPEB3-modulated genes using RT-qPCR. CPEB3 overexpression had insignificant effects on gene expression in HT22 cells. Notably, CPEB3 partially modulated differential gene splicing enhanced in the modulation of neural development, neuron cycle, neurotrophin, synapse, and specific development pathway, implying an alternative splicing regulatory mechanism associated with neurogenesis. Moreover, qRT-PCR verified the CPEB3-modulated transcription of neurogenesis genes LCN2 and NAV2, synaptogenesis gene CYLD, as well as neural development gene JADE1. Herein, we established that CPEB3 is a critical modulator of alternative splicing in neurogenesis, which remarkably enhances the current understanding of the CPEB3 mediated alternative pre-mRNA splicing.

## INTRODUCTION

The mammalian brain is a uniquely complex but a well-structured system, constituting various types of neuronal cells which form specific synaptic links with other neurons [1]. It is particularly essential to comprehend how neurogenesis, development, pathfinding, maintenance, migration, and synaptogenesis are modulated with such precision. Recently, alternative splitting decisions were thought to control key neuronal developmental phases, like neurogenesis, synaptic connectivity, plasticity, and remodeling [2–5]. Furthermore, a growing number of neurological illnesses (e.g., autism, depression, Parkinson's disease) correlated with known or suspected splicing deficiencies, suggests the potential significant function of neural alternative splicing events in several biological processes [6–8]. Recent advances have been made toward understanding the contributions of alternative splicing that modulate isoform switching during neurogenesis [9].

Neurogenesis is typified by extensive alterations in the transcriptomes, as well as proteomes of the differentiating cells, required for the optimal transformation of neural precursor or stem cells to mature neurons [2, 10]. Various gene modulatory pathways moderate the phases of neuronal growth consisting of neuronal relocation, neuronal plasticity, synaptic formations, dendritic and axonal outgrowth [2, 8]. The dynamic modulation of AS in the nervous system is essential for moderating protein-protein interactions, transcription systems, and neuronal growth [2].

Diverse mechanisms of spatio-temporal gene regulation have proved essential for the moderation of nervous system design, including control of mRNA synthesis via RNA-binding proteins (RBPs) [11]. Among these, cytoplasmic polyadenylation element-binding protein (CPEB) family is a crucial RNA-binding protein that has been noted in developing, synaptic plasticity and cellular senescence [12, 13]. CPEB confines the strength of glutamatergic synapses through regulating the translation of several plasticity-related proteins (PRPs) RNAs in neurons [14–16]. This RNA-binding protein controls the cytoplasmatic polyadenylation and translation of target mRNAs at synapses via a self-sustaining, functional prion-like shape [17]. This prion-like process shifts CPEB monomers from their original conformation into a differential, self-propagating shape, in which CPEB establishes continuously active build-ups [18–20]. In the vertebrates, four members (CPEB1, CPEB2, CPEB3, and CPEB4) have been identified, all expressed in the brain [21], and share the structure and sequence-specific identity in the RNA-binding domain [22]. CPEB1 achieves the translation of mRNA in target mRNA and subsequent binding of the cap-binding factor. Differently, CPEB2-4 has distinct U-rich loop motifs, suggesting targets unique to those subgroups [23]. Functionally, CPEB3 modulates the translation of several PRP RNAs, including NMDA receptor subunit 1 (NR1), AMPA-type glutamate receptor subunits GluA2, and GluA1, postsynaptic density protein 95 (PSD95), and the cytoskeletal protein actin [16, 24]. Particularly, CPEB3 allows a pathway for maintaining protein synthesis changes long after the initiating synaptic stimulus, a critical component, and a long-elusive piece in the puzzle of long-term memory maintenance [25]. Elevated calcium influx resulting from NMDA stimuli activates calpain-2 and then cleaves the CPEB3 repression motif to achieve non-polyadenylation activation of CPEB3-RNA targets [15]. Maintenance of translational control by CPEB3 guides the required protein modifications required for the establishment and maintenance of neuronal development and synaptosome [23]. These findings support the importance of CPEB3 in its regulatory function in neurons. As such, the RNA-binding protein CPEB3 mediates the translational activity of several identified mRNA targets in neurons [26, 27]. Several mechanisms modulate CPEB3-related translation in neuronal development, migration, and synaptogenesis [14, 28]. Nevertheless, whether CPEB3 modulates differential splicing of pre-mRNAs in the neuron cells remains unclear.

Here, we first cloned and excessively expressed CPEB3 in HT22 cells obtained from neurons in the hippocampus of mice. To establish the potential role of CPEB3 in modulating gene expression or alternative splicing (AS) that might be related to neurogenesis and neuronal development, we obtained CPEB3-regulated transcriptomes in mouse HT22 cells via RNA-seq. We then described the features of CPEB3 related differential gene expression and alternative splicing based on comparative analysis. Moreover, iRIP-seq was used to identify CPEB-binding targets in differential gene expression analysis and alternative splicing events. These findings indicate that CPEB3 modulates neurogenesis and neuronal development. Herein, we revealed that the RNA-binding protein CPEB3 is a crucial modulator of differential splicing in the central nervous system.

## RESULTS

### Profiling CPEB3-regulated gene expression

**Figure 1:**
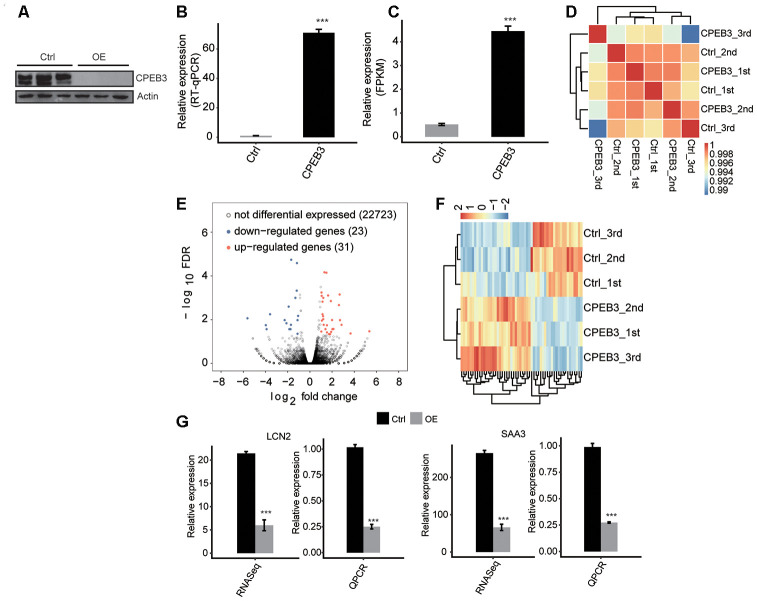
**CPEB3 overexpression has little effect on gene expression in HT22 cells.** (**A**) CPEB3 expression quantified by qRT-PCR. Error bars represent mean ± SEM. ***p < 0.001. (**B**) Western blotting analysis of CPEB3 expression. (**C**) CPEB3 expression quantified by RNA sequencing data. FPKM values were calculated as that has been explained in Materials and Methods. Error bars represent mean ± SEM. ***p < 0.001. (**D**) The heat map shows the hierarchically clustered Pearson correlation matrix resulted from comparing the transcript expression values for control and CPEB3 overexpression samples. (**E**) Identification of CPEB3 regulated genes. Up-regulated genes are labeled in red, whereas down-regulated are labeled in blue in the volcano plot. (**F**) Hierarchical clustering of DEGs in control and CPEB3 overexpression samples. FPKM values are log_2_-transformed and then median-centered by each gene. (**G**) Validation of gene expression of DEGs using qRT-PCR. RNA-seq quantification is shown at left, and RT-qPCR validation is shown at right.

To determine the molecular mechanism of CPEB3-mediated gene expression, we examined the model of CPEB3 overexpression (CPEB3-OE) in HT22 cells. Relative to the control group, the efficacy of CPEB3-OE was remarkably higher (Figure 1A). At the same time, the CPEB3-OE group had distinctly elevated protein levels (Figure 1B). To study the CPEB3-moderated transcriptional modulation, we prepared cDNA libraries of the control, as well as the CPEB3-OE cells. The sequencing of the six RNA-seq libraries was accomplished on the Illumina HiSeq4000 platform to generate 150nt paired-end reads per sample. The sequencing data were reviewed to validate their reliability (Supplementary Table 1). In comparing gene expression patterns across samples, the expression values (fragments/kilobase of exon model per million fragments mapped (FPKM)) were computed. Effective excessive expression of CPEB3 was moreover verified in a comparative RNA-seq assessment (Figure 1C). We utilized the FPKM values to compute an association matrix as per Pearson’s correlation coefficients. Consequently, the diagonal of the heat map revealed the Pearson association linking CEPB3-OE and control cells (Figure 1D). The biological replicates were highly associated.

### CPEB3 resulted in some transcriptional difference

The RNA-seq data acquired from the CEPB3-OE and from control cells was used to explore CPEB3-regulated genes at the transcriptional level. We employed the criteria of an absolute fold change ≥2 and FDR ≤0.05 with the edgeR package to determine the differentially expressed genes (DEGs) modulated by CPEB3 at the transcriptional level (Supplementary Table 2). Consequently, 31 up modulated and 23 down modulated genes related with CPEB3 were identified. We constructed a volcano plot to show the remarkably expressed genes correlated with CPEB3-OE (Figure 1E). A heat map estimation of the DEG expression trends in the RNA-seq samples showed high consistency with the CPEB3-regulated transcription in both datasets (Figure 1F).

It was possible that the activated expression of the neurogenesis genes was by CPEB3 excessive expression. Therefore, we conducted a qPCR analysis to validate the RNA-seq results. To examine the possibility, we assessed the levels of Lipocalin-2 (LCN2) and serum amyloid A3 (SAA3) mRNA expression. These genes were highly expressed in neurogenesis, neuronal development, and neuroinflammation. We demonstrated a significant increase in LCN2 and SAA3 expression, highly consistent with the sequencing data (Figure 1G).

### CPEB3 overexpression modulates pre-mRNA alternative splicing of neurons and mediates neurogenesis-related genes

**Figure 2:**
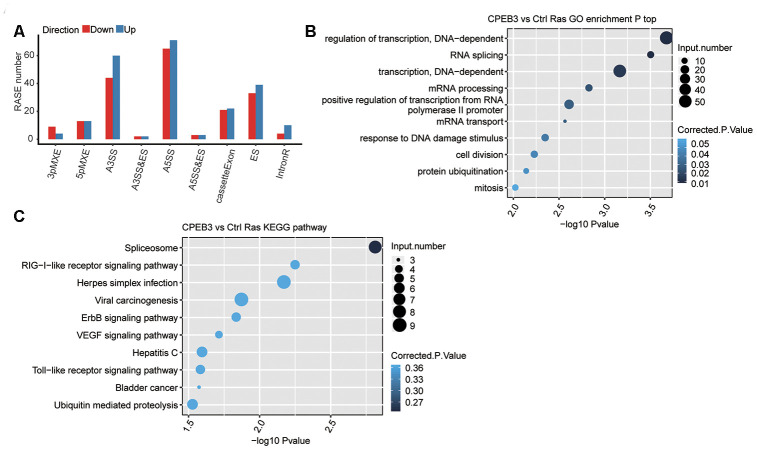
**RNA-seq data reveals CPEB3 regulates gene alternative splicing in HT22 cells.** (**A**) Classification of CPEB3 overexpression regulated alternatively spliced events. (**B**) The top 10 enriched GO biological processes of the CPEB3-regulated alternatively spliced genes. (**C**) The top 10 enriched KEGG pathways of the CPEB3-regulated alternatively spliced genes.

One primary aim of this study constituted gaining insights into the function of CPEB3 on alternative splicing regulation. To assess the CPEB3-dependent AS effects in HT22 cells, we evaluated the data quality using an analysis of differential splicing. A total of 93M ± 6.9M uniquely mapped reads were retrieved from CPEB3-OE and control HT22 cells, in which about 47.62%~ 48.83% were junction reads (Supplementary Table 1). Analysis of the annotation of the reference genome identified 170,257 novel splice junctions using the Tophat2 pipeline), 141,720 annotated splice junctions and 68.03% annotated exons (187,900 out of 284,564 annotated exons) (Supplementary Table 3). Subsequently, we employed the ABLas software [29] to assess the AS events from the RNA-seq dataset to determine the global variations in the AS profiles in response to CPEB3-OE. We detected 167,859 novel ASEs, excluding intron retention (IR) and 35,128 known ASEs in the model gene named in the reference genome (Supplementary Table 3). Using a stringent cutoff of p-value ≤0.05, changed AS ratio ≥ 0.2, we identified high-confidence RASEs, which gave rise to 497 RASEs (Supplementary Table 4). Complete RASEs included 82 known intron-retention (IR) RASEs and 415 non-IR (NIR) RASEs. The NIR RASEs constituted 104 alternative 3’ splice site (A3SS), 136 alternative 5’ splice site (A5SS), 72 exons skipping (ES), and cassette exon (CE, 43) (Figure 2A). The data indicated that CPEB3 modulates ASEs in HT22 cells globally.

In the biological process terms of GO assessments, the up modulated genes A large number of genes with alternative splicing in the CPEB3 overexpressed cells were enriched in cell proliferation and transcription, including cell division, mitosis, regulation of transcription, RNA splicing, transcription (Figure 2B). Enriched KEGG pathways included VEGF, RIG-I-like receptor, and Toll-like receptor signaling pathways (Figure 2C, Supplementary Table 5). These findings posit that CPEB3 potentially plays a critical role in neurodevelopment and immunity.

### Validation of CPEB3-regulated AS events in HT22 cells

**Figure 3:**
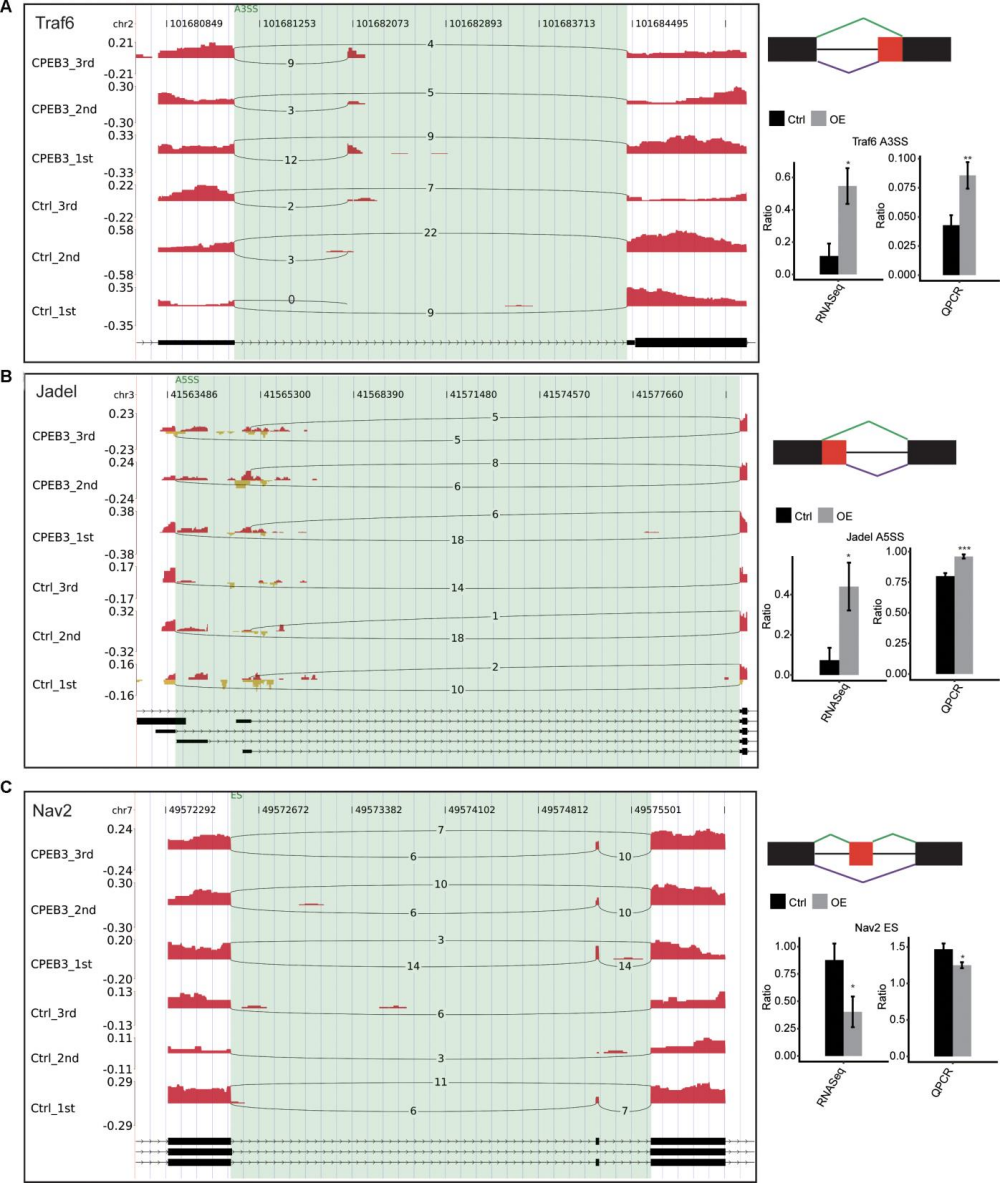
**Validation of CPEB3 regulated AS events.** (**A**–**C**) IGV-sashimi plot showed an alternative 3’ splicing sites (**A**), an exon skipping (**B**), and a cassette exon (**C**) events in three different genes. Reads variations on the alternative exon were plotted in the left panel with the transcripts shown below. The schematic diagrams depict the structures of ASEs, AS1 (purple line), and AS2 (green line). The constitutive exon sequences are denoted by black boxes, intron sequences by a horizontal line (right panel, top), while alternative exon by the red box. RNA-seq quantification and RT-qPCR validation of ASEs are shown at the bottom of the right panel. Error bars represent mean ± SEM. *p < 0.05.

To confirm the differential splicing effects determined using the RNA-Sequencing data, we evaluated potential differential splicing events using qPCR. We designed the PCR primer pairs for multiplexing the long and short splicing isoforms (Supplementary Table 6). The differential splicing events verified by the qPCR data were consistent with the RNA-Seq data, located in tumor necrosis factor receptor-associated factor 6 (TRAF6), JADE1, and neuron navigator 2 (NAV2). We found a significant increase in TRAF6 and JADE1. There were distinct reductions in NAV in the CPEB-OE cell lines. All the results were consistent with the RNA-seq analysis. Most of these genes are involved in neuronal development, neuron progenitors, or neuroinflammation directly or indirectly involved in neurological disorders (Figure 3). The findings validated the CPEB3-modulated ASEs determined by ABLas assessments of RNA-seq.

### CPEB3-mRNA interaction map in HT22 cells

**Figure 4:**
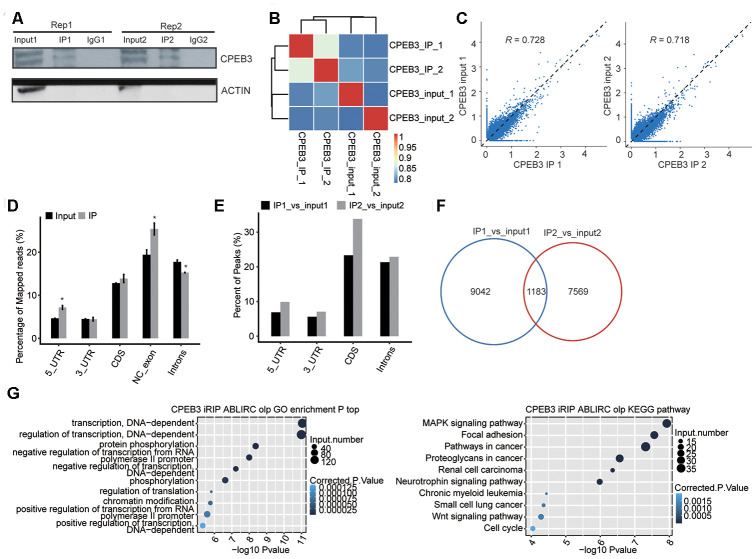
**RIP-seq analysis of CPEB3.** (**A**) Western blotting analysis of immunoprecipitated CPEB3 from HT22 cells. (**B**) The heat map shows the hierarchically clustered Pearson correlation matrix resulted from comparing the transcript abundance between anti-flag and IgG immunoprecipitated samples. (**C**) Scatter plot of transcript abundance across the reference genome in paired samples. (**D**) Reads distribution across the reference genome. Error bars represent mean ± SEM. *p < 0.05. (**E**) Peak distribution across different genomic regions. (**F**) Venn diagram of peaks in two replicates. (**G**) The top 10 enriched GO biological processes(left) and KEGG pathways(right) of the CPEB3-bound genes.

To establish the correlation of CPEB3-mRNA interaction in HT22 cells, we constructed a transcriptome-wide binding profile of CPEB3 using the iRIP-seq approach [30]. iRIP captures both direct and indirect RNA–RBP interactions. The flag-tagged CPEB3 was employed for immunoprecipitation, and two separate iRIP replicates were conducted. Flag-CPEB3 protein was identified through western blotting of both total cell lysate and Flag IP samples, but not in the IgG control (Figure 4A). We sequenced the cDNA libraries from anti-Flag IP and the complete cell lysate control on an Illumina X-ten platform. The heat map revealed the hierarchically classified Pearson association matrix generated from comparing the transcript abundance between anti-flag and IgG immunoprecipitated samples (Figure 4B). A plot of the assayed levels of each gene was reflected using the FPKM in each pair of the samples, which suggested that transcripts were enriched in the IP samples (Figure 4C). Reads distribution across reference genome revealed the Flag-CPEB3 reads were statistically enhanced in gene sites consisting of the 5’UTR, noncoding (NC) exons, and introns, when contrasted with the input control reads (Figure 4D). Peak distribution across different genomic regions revealed the IP group was enhanced in gene regions consisting of the 5’UTR, 3’UTR, CDS, and introns (Figure 4E). The peaks were identified using the ABLIRC algorithm. Peaks from the two sets of experiments overlapped well (Figure 4F). It further indicated that these genes interacting with CPEB3 were highly enhanced for modulation of transcription, protein phosphorylation, and regulation of translation (Go biological process terms, Figure 4G, left panel). Enriched KEGG cascades constituted MARK signaling cascade, focal adhesion, neurotrophin signaling cascade, Wnt signaling axis, and the cell cycle (Figure 4G, right panel, Supplementary Table 7). The CPEB3-bound genes were enriched in transcription, neurodevelopment, and neurogenesis genes.

### Functional analysis and validation of the CPEB3-bound genes in HT22 cells

**Figure 5:**
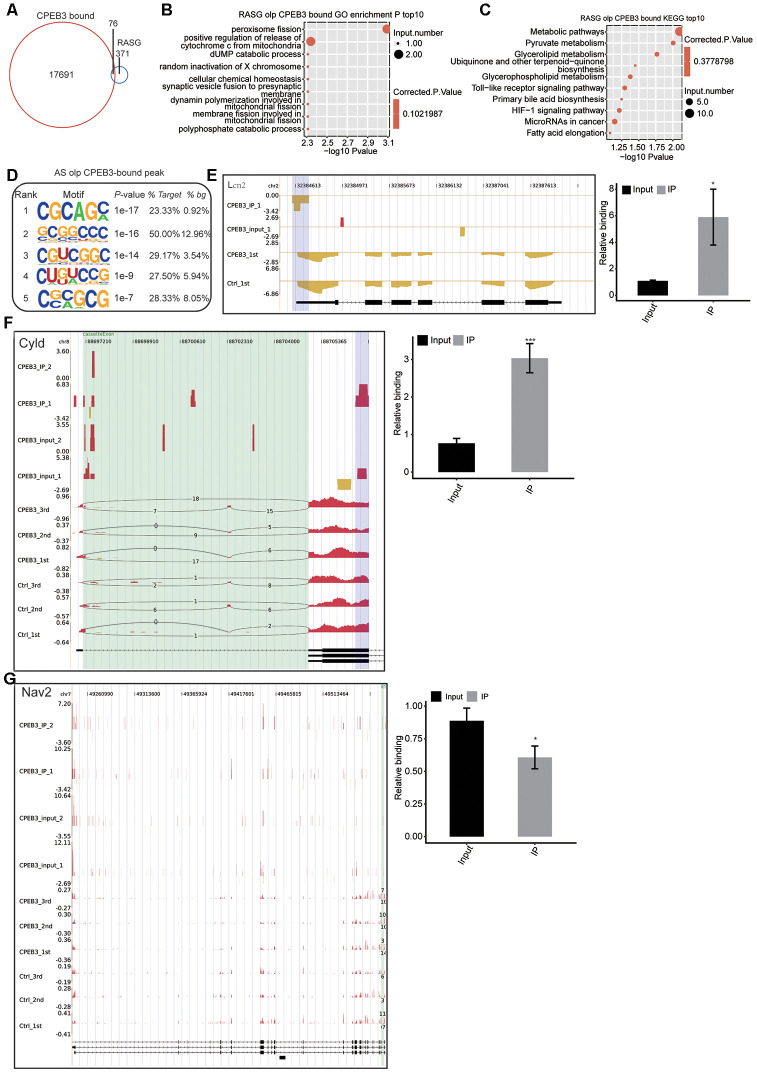
**Integrated analysis of CPEB3-bound genes and the regulated alternative splicing events (RASE) in response to CPEB3 overexpression.** (**A**) The overlap of CPEB3-bound peaks with CPEB3-regulated alternative splicing events. (**B**)Top 10 GO biological process in which the overlapped genes in A were enriched. (**C**) Top 10 KEGG pathways in which the overlapped genes in A were enriched. (**D**) The CG motifs over-represented in CPEB3 peaks overlapped with RASEs. The motifs were searched by running HOMER pipeline. (**E**–**G**) The reads density landscape of CPEB3-binding peaks on DEGs and RASEs (left). RIP-qPCR validation of the CPEB3 binding a peak was showed (right). The asterisk (*) indicates *P < 0.05,** P < 0.01.

We used the ABLIRC tool to elucidate the CPEB3-bound genes from the iRIP-seq reads. Consequently, 76 of the CPEB3-bound genes (17691) overlapped with the CPEB3-regulated alternative splicing genes (371) (Figure 5A), indicating that CPEB3 could modulate differential splicing processes via direct binding to RNA targets. We overlapped the CPEB3-bound genes with the RASG. Go enrichment to determine the CPEB3 binding related differential splicing modulation. This further revealed that the CPEB3-modulated genes are involved in synaptic formation (Figure 5B). Enriched KEGG pathways were enriched for metabolic pathways, pyruvate metabolism, and Toll-like receptor signaling pathway (Figure 5C). We found that the GC-rich motifs were highly enhanced in the AS overlapped CPEB bound peak (Figure 5D), suggesting that the first GC in the motif were the critical sites for CPEB3 binding to its targets. We performed qPCR analyses to validate the CPEB3-bound genes overlapping with RASG detected in this study. Lcn2 was markedly enriched in the anti-CPEB3 immunoprecipitate contrasted with the input. The association of Lcn2 mRNA to CPEB3 was besides confirmed by the qPCR assays (Figure 5E). Notably, both the RNA-seq, as well as the iRIP-seq peaks posited that Lcn2 is a direct target of CPEB3 protein in HT22 cells. Similarly, NAV2 was distinctly lower in the anti-CPEB3 immunoprecipitate contrasted with the input, in agreement with RNA-seq data (Figure 5G). Moreover, CPEB3 led a significant increase of cylindromatosis (CYLD) in the CPEB3-OE IP arm, which was confirmed by the qPCR assay (Figure 5F). This result indicated that CPEB3-mediated alternative splicing of CYLD is involved in synaptogenesis. Collectively, we concluded that CPEB3 is preferentially bound to neurogenesis and synaptogenesis genes.

## DISCUSSION

The RNA-binding protein CPEB3 mediates the translational activity of several identified mRNA targets in neurons. However, the regulatory mechanism through which CPEB3 mediates translation remains unknown. To our best knowledge, we first established that CPEB3 broadly modulates the differential splicing of genes that participate in neurogenesis in HT22 cells. Herein, we utilized RNA-seq and iRIP-seq strategy to elucidate the CPEB3-RNA interactions in neuron cells globally. It involved 31 up modulated genes and 23 down modulated genes, posting that CPEB3 has an insignificant impact on gene expression in HT22 cells. Interestingly, we found that the overexpression of the CPEB3 resulted in elevated LCN2 mRNA levels in HT22 cells, which suggested that CPEB3 modulates the splicing trend of LCN2 pre-mRNA. LCN2 is an alternative mechanism for the delivery and absorption of physiological iron [31]. Recently, LCN2 was reported to possess various roles in cell iron transportation and homeostasis [32, 33]. Within the brain, the iron-binding potential of LCN2 was proved to be significant in regulating hippocampal neuronal dendritic spine density and morphology [34]. Importantly, LCN2 is also a critical modulator of neurogenesis that regulates the neural stem cells (NSCs) maintenance, self-renewal, proliferation, differentiation, and hippocampal plasticity and function [35]. Moreover, the iRIP-seq analysis showed that CPEB3 preferentially binds LCN2. Similarly, SAA3, an acute-phase protein with a cytokine-like feature, was elevated in CPEB-OE cell lines. SAA3 is a crucial modulator in neuronal survival and death during inflammation [36]. These findings contribute to the existing knowledge on the mechanisms that modulate neurogenesis and neuronal development.

Evaluation of RNA-seq data obtained in the present study showed that CPEB3 globally modulates AS in HT22 cells. Particularly, CPEB3 binding is linked to increased RASE numbers. In the functional assessments, these changes in gene expression impacted cell division, transcription regulation, mitosis, and multiple signaling pathways. Specifically, CPEB3 correlated with the RIG-I-like receptor, Toll-like receptor, and VEGF signaling cascade. Recent mounting evidence posited that VEGF supports neuronal progenitors as they proliferate, migrate, and differentiate [37]. Our findings provide comprehensive insights on the differential splicing modulation mechanism of CPEB3 as neuronal development and neurogenesis.

In particular, the broad significance of AS events occurring involves the modulation of transcription. For instance, the genes constitute TRAF6, JADE1, and NAV2. TRAF6 has significantly been reported to modulate neuronal fate [38]. Previous studies suggested that JADE1 links cell development, cell cycles, DNA transcription, and replication hence participating in epigenetic processes [39]. Jade1 is also subject to posttranscriptional regulation, resulting in various transcripts, which mediate a regulated expression during cell cycle progression [40]. The CPEB3-OE HT22 cells exhibit high expression levels of NAV2, which play a role in nervous system development, including neurite outgrowth, axonal elongation, neuronal proliferation, and migration [41, 42]. Notably, CPEB3 had similar effects on NAV2 through the iRIP-seq analysis.

Overall, the iRIP-seq approach identifies the intact RNA biomolecules bound by RBPs. Herein, we utilized the ABLIRC algorithm to effectively determine binding peaks and motifs of CPEB3 protein from iRIP-seq results. iRIP-seq elucidated some indirect CPEB3 binding regions in the 5’ UTR sites, and likely in the introns and NC-exon regions. CPEB3 enriched-regulation of binding genes in the regulation of translation, regulation of transcription, MAPK signaling cascade, focal adhesion, neurotrophin signaling cascade, Wnt signaling cascade, and the cell cycle. Therefore, our study expands current knowledge of the primary role of CPEB3 in the regulation of neurogenesis.

Overall, 35,128 alternative splicing events were identified, and 447 RASEs were detected. Among them, 76 of the pre-mRNAs were modulated by the direct binding of CPEB3 in HT22 cells. Furthermore, we established that differential splicing modulation of CPEB3 is global, which makes RNA-binding protein versatile in the regulation of various alternative splice events. When CPEB3-bound peaks overlapped with RASG events, synaptic vesicle fused to presynaptic membrane, pyruvate metabolism, and the toll-like receptor. The CPEB-binding genes in these pathways included LCN2, CYLD, and NAV2. LCN2 and NAV2 have been well-established to contribute to neurogenesis and neuronal development. Our results regarding CPEB3-modulated differential splicing of LCN2, as well as NAV2, revealed a more multiplex network in neurogenesis. CYLD, a deubiquitinase distinct for K63-linkage polyubiquitins, is highly expressed in the brain. CYLD has been reported as one of the abundant proteins in the affinity-purified PSD fraction [43]. In addition, CYLD modulates dendritic growth and postsynaptic differentiation in mouse hippocampal neurons [44]. These results revealed that CPEB3-mediated alternative splicing of CYLD is potentially involved in synaptogenesis.

## CONCLUSION

Herein, for the first time, we established that CPEB3 broadly modulates the differential splicing of genes that participate in neurogenesis in HT22 cells. The direct modulation of the gene expression enhanced in neuroinflammation and synaptogenesis has also been demonstrated. More studies should be conducted to explore the biological role of CPEB3-modulated neurite outgrowth, axonal elongation, synapse formation, and neuroinflammation. Hence, we provide new insights that CPEB3 potentially participate in AS events, necessary for the discovery of novel biological roles of CPEB3 in the process of neurogenesis and neural development.

## MATERIALS AND METHODS

### Cloning and plasmid construction

We employed CE Design V1.04 (Vazyme, Nanjing, China) to design the primer pairs utilized for Hot Fusion. Every primer includes gene-distinct fragment sequence and a 17-30 bp pIRES-hrGFP-1a vector sequence.

F-primer: agcccgggcggatccgaattcATGCAGGATGATTTACTGATG R-primer: gtcatccttgtagtcctcgagGCTCCAGCGGAACGGGAC Using EcoRI and XhoI (NEB), we digested the pIRES-hrGFP-1a vector at 37° C for 2h~3h. Then, the vector digested by the enzyme gel-electrophoresed on 1.0% agarose gel, followed by purification employing the Qiagen column kit (Qiagen. Inc, Valencia, CA). Using Trizol reagent (15596, Life Technologies, USA), we extracted total RNA from the HT22 cells. Subsequently, we reverse-transcribed the RNA to cDNA using an oligo-dT primer. After that, the insert fragment using PCR, we detected the insert fragment. Ligation of the vector that was linearized using digestion with EcoRI and XhoI (NEB) and the PCR insert was accomplished using ClonExpress® II One Step Cloning Kit (Vazyme). Through chemical transformation, we introduced the plasmids into the *Escherichia coli* strain. Subsequently, the cells were grown on LB agar plates added gL/ml ampicillin in an overnight incubation at 37° C. We screened the colonies through colony PCR (28 cycles) using universal primers (positioned on the backbone vector). The inserts sequence were Sanger sequenced for verification.

### Assessment of CPEB3 overexpression

We utilized the GAPDH (glyceraldehyde-3-phosphate dehydrogenase) gene as our standard control to examine the impacts of excessive expression of CPEB3. cDNA generation was accomplished through standard procedures, and RT-qPCR using the Bestar SYBR Green RT-PCR Master Mix (2220, DBI Bioscience, Shanghai, China) run on the Bio-Rad S1000 machine. Using the 2-AACT approach, we normalized the concentrations of our assay transcripts to the GAPDH mRNA levels [45]. Comparisons were accomplished with the Student's paired t-test in the GraphPad Prism software (San Diego, CA, USA).

### RNA extraction and sequencing

Before RNA extraction, the HT22 cells were made into a fine powder via grinding. We utilized the TRIZOL reagent for the RNA isolations. After that, we performed two phenol-chloroform treatments to remove contaminating biomolecules and salts from the RNA isolates, followed by the addition of RQ1 DNase (Promega, Madison, WI, USA) to digest any contaminating DNA. We checked the quality and quantity levels of the purified RNA by assessing their absorbance at 260/280nm (A260/A280) on a Smartspec Plus (BioRad, Hercules, CA, USA). Moreover, we gel-electrophoresed the purified RNA on a 1.5% agarose gel to check its integrity.

For each sample, we used 1ng of the RNA in preparing the RNA-seq library using the VAHTS Stranded mRNA-seq Library Prep Kit (NR604, Vazyme, Nanjing, China). Subsequently, we purified the polyadenylated mRNAs, and then fragmented them, followed by the generation of double-strand cDNA. We then ligated the ds-cDNAs to the VAHTS RNA Adapters (N803/N804, Vazyme, Nanjing, China) following the step of end repair + A tailing. The purified ligation products (size 200-500 bps) were cleaved using the heat-labile UDG. We then amplified the resulting single-strand cDNAs, which were purified, quantified, and kept at -80° C, waiting for sequencing.

For high-throughput sequencing, we performed library preparation as outlined by the manufacturer and conducted on an Illumina HiSeq X Ten system to generate 150-nt paired-end products.

### RNA-Seq raw data cleaning and alignment

Firstly, we dropped the raw reads with more than 2-N bases. After that, we implemented an adaptor and low-quality base trimming from raw sequenced reads in FASTX-Toolkit (Version 0.0.13). Subsequently, we dropped short reads below 16nt. After that, mapping of the trimmed clean reads to the GRch38 genome using tophat2, allowing four mismatches, was performed [46]. We applied the uniquely mapped reads for gene reads number counting and FPKM calculation (fragments per kilobase of transcript per million fragments mapped) [47].

### RNA-Seq and differentially expressed genes (DEG) evaluation

The R Bioconductor package edgeR was used to screen out the DEGs [48]. We set a false discovery rate <0.05 and fold change>2 or < 0.5 as the cutoff values for identifying DEGs.

### RNA-Seq and differential splicing assessment

The differential splicing events (ASEs) and regulation-related differential splicing events (RASEs) between these samples were identified and quantified via the ABLas pipeline, as outlined previously [49]. In short, the identification of ten kinds of ASEs was as per the splice junction reads, i.e., exon skipping (ES), alternative 5' splice site (A5SS), alternative 3'splice site (A3SS), intron retention (IR), mutually exclusive exons (MXE), mutually exclusive 5'UTRs (5pMXE), mutually exclusive 3'UTRs (3pMXE), cassette exon, A3SS&ES, and A5SS&ES.

To examine RBP modulated ASE, we performed the student's t-test to inspect the importance of the ratio alteration of AS events. Those events, which were remarkable at P-value cutoff correlated with a false discovery rate cutoff of 5%, were regarded to be RBP modulated ASEs.

### Reverse transcription qPCR verification of DEGs and AS events

To establish the validity of the RNA-seq data in HT22 cells, we conducted qRT-PCR of the selected DEGs. We used the remaining total RNA from RNA-seq library preparation for the RT-qPCR. First, we generated cDNA via reverse transcription using an M-MLV Reverse Transcriptase (R021, Vazyme, Nanjing, China). After that, we performed qPCR using the SYBR Green PCR Reagents Kit (QR0100, Yeasen, Shanghai, China) on Step One Real Time PCR System. The PCR conditions constituted denaturing at 95° C for 10min, 40 cycles involving denaturing at 95° C for 15s, annealing at 60° C for 1min, and extension at 60° C for 1min. Each sample was amplified in triplicates. We used the GAPDH gene as the standard to normalize the RNA expression levels of all the genes.

Meanwhile, the qRT-PCR assay was also used for ASE verification. The primers for identifying ASEs are itemized in Additional file 1. To identify alternative isoforms, we utilized a boundary-spanning primer for the sequence comprising of the junction of the constitutive exon, alternative exon, and an opposing primer in a constitutive exon. The boundary-spanning primer of alternative exon was developed as per the model exon to identify model splicing or altered exon to identify differential splicing.

The Primers we used herein are presented in the Additional file 1.

### Co-Immunoprecipitation

Firstly, we lysed the HT22 in ice-chilled lysis buffer (IxPBS, 0.5% sodium deoxycholate, 0.1% SDS, 0.5% NP40) with RNase repressor (Takara, 2313) and a protease suppressor (Solarbio, 329-98-6) on ice for 5min. Subsequently, the mixture was vibrated vigorously, followed by centrifugation at 13,000 x g at 4° C for 20min to eliminate the cell debris. We incubated the supernatant with DynaBeads protein A/G (Thermo, 26162) conjugated with anti-flag antibody (Sigma, F1804) or normal IgG at 4° C for the overnight. The beads were rinsed with Low-salt Wash buffer, High-salt Wash buffer, and 1X PNK Buffer, respectively. We resuspended the beads in the elution buffer, followed by dividing into two classes, one for RNA extraction from CPEB3-RNA complexes and another for the western blotting assay for CPEB3.

### Western blot

We resuspended the samples in 40μl elution Buffer (50mM Tris-Cl (PH=8.0), 10mM EDTA (PH=8.0), 1%SDS, followed by incubation at 70° C for 20min at 1,400 rpm. After that, the samples were centrifuged at 13,200xg for a short time period. We then moved the supernatant to a fresh EP tube while on the magnetic separator. After the complexes were eluted by boiling for about 10min in boiling water containing 1X SDS sample buffer, the proteins were resolved on 10% SDS-PAGE, with TBST buffer (20 mM Tris-buffered saline and 0.1% Tween-20) added 5% non-fat milk powder for 1h at RT. We incubated the membranes with the primary antibody: Flag antibody (1:2,000, Sigma, F7425), actin (1:2000, CUSABIO), followed by HRP-conjugated secondary antibody. The conjugated secondary antibody (anti-mouse or anti-rabbit 1:10,000) (Abcam) was measured using the enhanced chemiluminescence (ECL) reagent (Bio-Rad, 170506).

### iRIP-Seq library preparation and sequencing

The CPEB3-bound RNAs were extracted from the immunoprecipitation of anti-Flag using the TRIzol reagent (15596-026, Invitrogen, NY, USA). cDNA libraries were built using the KAPA RNA Hyper Prep Kit (KAPA, KK8541) as per the protocol outlined by the manufacturer. High-throughput sequencing of the cDNA libraries was accomplished on an Illumina Xten platform for 150 bp paired-end sequencing.

### Data analyses

After aligning the reads onto the genome using TopHat 2 [46], we only utilized the uniquely mapped reads in the subsequent analysis. We used the ABLIRC approach to establish the binding sites of CPEB3 on the genome [29]. The reads with at least 1bp overlap were grouped as peaks. For every gene, we applied computational simulation in randomly generating reads with similar numbers and lengths as reads in peaks. We further mapped the outputting reads to the same genes; hence, we generated random max peak height from the overlapping reads. We repeated the whole process 500 times. We selected all the reported peaks with heights higher relative to the random max peaks (p-value < 0.05). The IP and input samples were assessed using the simulation separately, with the IP peaks that had overlaps with Input peaks removed. Finally, the target genes of IP were established using the peaks and the binding motifs of IP protein were named using the HOMER software (Heinz, Benner et al. 2010).

### Functional enrichment estimations

To categorize the functional groups of peak associated genes (target genes), Gene Ontology (GO) terms and KEGG pathways were elucidated using the KOBAS 2.0 server [50]. We utilized the Hypergeometric test and Benjamini-Hochberg FDR controlling protocol to describe the enrichment of each term.

### Availability of data and materials

The data generated and discussed in this publication are available under GEO Series accession (GSE153168).

## Supplementary Material

Supplementary Table 1

Supplementary Table 2

Supplementary Tables 3 and 4

Supplementary Table 5

Supplementary Table 6

Supplementary Table 7

Additional File 1
