# Supplementary material for: CPEB3 regulates neuron-specific alternative splicing and involves neurogenesis gene expression
Source: Aging (Albany NY). 2020 Dec 9;13(2):2330–47. doi: 10.18632/aging.202259 (PMC7880327; doi:10.18632/aging.202259)
Supplement: Supplementary Table 2 [file aging-13-202259-s002.docx]

**Supplementary Table 2. CPEB3-OE versus Ctrl DEGs.**

| **Gene** | **logFC** | **logCPM** | **PValue** | **FDR** | **CPEB3**  **_1st** | **CPEB3**  **_2nd** | **CPEB3**  **_3rd** | **Ctrl**  **_1st** | **Ctrl**  **_2nd** | **Ctrl**  **_3rd** | **up/**  **down** | **Symbol** | **Chromosome** | **Genetype** | **Description** |
| --- | --- | --- | --- | --- | --- | --- | --- | --- | --- | --- | --- | --- | --- | --- | --- |
| ENSMUSG00000039652.14 | 3.076280111 | 4.157149085 | 2.83E-54 | 6.44E-50 | 3.94 | 4.42 | 4.99 | 0.64 | 0.47 | 0.45 | up | Cpeb3 | chr19 | protein_coding | cytoplasmic polyadenylation element binding protein 3 |
| ENSMUSG00000040026.7 | -2.035475265 | 6.54679001 | 7.08E-29 | 8.06E-25 | 76.93 | 71.93 | 49.37 | 254.95 | 279.29 | 261.03 | down | Saa3 | chr7 | protein_coding | serum amyloid A 3 |
| ENSMUSG00000101249.1 | -2.196733166 | 2.868532509 | 1.23E-28 | 9.35E-25 | 5.97 | 4.24 | 5.07 | 4.45 | 60.78 | 3.62 | down | Gm29216 | chr1 | unprocessed_pseudogene | predicted gene 29216 |
| ENSMUSG00000026822.12 | -1.873226883 | 3.738304144 | 2.83E-23 | 1.61E-19 | 8.06 | 5.88 | 4.03 | 20.67 | 22.05 | 21.58 | down | Lcn2 | chr2 | protein_coding | lipocalin 2 |
| ENSMUSG00000020826.6 | -1.783805032 | 3.691751289 | 2.47E-21 | 1.13E-17 | 1.68 | 1.35 | 0.96 | 5.13 | 4.88 | 3.5 | down | Nos2 | chr11 | protein_coding | nitric oxide synthase 2, inducible |
| ENSMUSG00000092837.1 | -1.73560427 | 3.499357023 | 3.48E-20 | 1.32E-16 | 10.43 | 10.15 | 27.01 | 13.73 | 19.04 | 114.1 | down | Rpph1 | chr14 | ribozyme | ribonuclease P RNA component H1 |
| ENSMUSG00000064246.8 | -1.685708341 | 2.999544503 | 1.69E-18 | 5.50E-15 | 1.88 | 2.04 | 1.7 | 5.76 | 6.34 | 5.57 | down | Chil1 | chr1 | protein_coding | chitinase-like 1 |
| ENSMUSG00000055030.1 | -1.681987327 | -0.439882123 | 6.41E-09 | 1.83E-05 | 0.51 | 0.59 | 0.39 | 1.19 | 1.48 | 1.98 | down | Sprr2e | chr3 | protein_coding | small proline-rich protein 2E |
| ENSMUSG00000029371.7 | -1.167471112 | 1.71182044 | 1.02E-08 | 2.59E-05 | 1.24 | 0.99 | 1.45 | 3.38 | 2.92 | 1.82 | down | Cxcl5 | chr5 | protein_coding | chemokine (C-X-C motif) ligand 5 |
| ENSMUSG00000070828.7 | 1.307343915 | 0.65605357 | 3.01E-08 | 6.85E-05 | 1 | 0.62 | 1.09 | 0.38 | 0.32 | 0.37 | up | Zscan4f | chr7 | protein_coding | zinc finger and SCAN domain containing 4F |
| ENSMUSG00000096879.2 | 1.488061259 | -0.022967103 | 3.45E-08 | 7.14E-05 | 0.48 | 0.53 | 0.83 | 0.31 | 0.11 | 0.22 | up | Gm4858 | chr3 | protein_coding | predicted gene 4858 |
| ENSMUSG00000023992.12 | -1.188123092 | 0.660396174 | 2.70E-07 | 0.000473712 | 1.04 | 0.64 | 0.71 | 1.45 | 2.08 | 1.75 | down | Trem2 | chr17 | protein_coding | triggering receptor expressed on myeloid cells 2 |
| ENSMUSG00000093489.1 | 1.043643208 | 1.759263206 | 3.52E-07 | 0.000573183 | 6.3 | 4.96 | 7.28 | 3.79 | 1.89 | 3.11 | up | Gm20625 | chr10 | lincRNA | predicted gene 20625 |
| ENSMUSG00000083396.1 | 2.679477156 | -2.122396163 | 4.65E-07 | 0.00070684 | 0.24 | 0.12 | 0.39 | 0.04 | 0.07 | 0 | up | Gm15542 | chr7 | processed_pseudogene | predicted gene 15542 |
| ENSMUSG00000079029.2 | 1.639652122 | -0.905407084 | 5.87E-07 | 0.00078646 | 0.27 | 0.36 | 0.5 | 0.08 | 0.15 | 0.12 | up | Gm5662 | chr12 | protein_coding | predicted gene 5662 |
| ENSMUSG00000037416.10 | 1.056838652 | 1.385684758 | 6.44E-07 | 0.000814536 | 0.2 | 0.17 | 0.42 | 0.2 | 0.11 | 0.07 | up | Dmxl1 | chr18 | protein_coding | Dmx-like 1 |
| ENSMUSG00000031722.9 | -1.307104639 | -0.097788991 | 8.73E-07 | 0.001007286 | 0.39 | 0.39 | 0.33 | 0.75 | 1.07 | 0.86 | down | Hp | chr8 | protein_coding | haptoglobin |
| ENSMUSG00000054272.5 | 1.206493179 | 0.374469624 | 9.29E-07 | 0.001007286 | 0.54 | 0.7 | 0.92 | 0.58 | 0.23 | 0.13 | up | Zscan4c | chr7 | protein_coding | zinc finger and SCAN domain containing 4C |
| ENSMUSG00000054555.9 | 1.888131042 | -1.37582414 | 1.37E-06 | 0.001413769 | 0.05 | 0.07 | 0.07 | 0.02 | 0.01 | 0.03 | up | Adam12 | chr7 | protein_coding | a disintegrin and metallopeptidase domain 12 (meltrin alpha) |
| ENSMUSG00000096175.3 | 1.139888784 | 0.522324954 | 1.54E-06 | 0.001509882 | 1.16 | 1.59 | 1.22 | 0.47 | 0.61 | 0.66 | up | Gm21761 | chr13 | protein_coding | predicted gene, 21761 |
| ENSMUSG00000104460.1 | 2.616425382 | -2.162656936 | 2.37E-06 | 0.0021562 | 0.35 | 0.21 | 0.25 | 0.09 | 0.02 | 0.02 | up | Gm9121 | chr3 | unprocessed_pseudogene | predicted pseudogene 9121 |
| ENSMUSG00000094377.1 | -1.914278508 | -1.667951153 | 2.90E-06 | 0.002536486 | 0.83 | 0.43 | 1.1 | 0.68 | 2.55 | 5.4 | down | Gm24407 | chr5 | snRNA | predicted gene, 24407 |
| ENSMUSG00000019929.13 | -3.588297995 | -2.81441534 | 7.65E-06 | 0.005662573 | 0.02 | 0 | 0 | 0.05 | 0.09 | 0.13 | down | Dcn | chr10 | protein_coding | decorin |
| ENSMUSG00000090714.7 | 1.139149999 | 0.039632581 | 7.71E-06 | 0.005662573 | 0.74 | 0.45 | 0.5 | 0.2 | 0.36 | 0.18 | up | Zscan4d | chr7 | protein_coding | zinc finger and SCAN domain containing 4D |
| ENSMUSG00000097296.1 | -1.061060599 | 0.384709574 | 9.45E-06 | 0.006727234 | 1.72 | 0.97 | 0.76 | 2.23 | 2.87 | 1.92 | down | Gm26532 | chr8 | lincRNA | predicted gene, 26532 |
| ENSMUSG00000070619.5 | 1.188016839 | -0.09353068 | 1.01E-05 | 0.00696205 | 0.56 | 0.54 | 0.59 | 0.38 | 0.19 | 0.17 | up | Gm13119 | chr4 | protein_coding | predicted gene 13119 |
| ENSMUSG00000002944.13 | -5.6264103 | -3.244495021 | 1.29E-05 | 0.008657494 | 0 | 0 | 0 | 0.05 | 0.04 | 0.02 | down | Cd36 | chr5 | protein_coding | CD36 antigen |
| ENSMUSG00000086728.2 | 1.202172661 | -0.278721333 | 1.46E-05 | 0.009481966 | 0.43 | 0.83 | 0.36 | 0.11 | 0.22 | 0.34 | up | Man2c1os | chr9 | antisense | mannosidase, alpha, class 2C, member 1, opposite strand |
| ENSMUSG00000085024.1 | 1.999724641 | -1.795096853 | 1.65E-05 | 0.010433474 | 0.42 | 0.33 | 0.44 | 0.16 | 0.04 | 0.09 | up | C230035I16Rik | chr13 | lincRNA | RIKEN cDNA C230035I16 gene |
| ENSMUSG00000020010.7 | -2.277766782 | -2.165829138 | 1.79E-05 | 0.010537696 | 0 | 0.1 | 0 | 0.19 | 0.19 | 0.11 | down | Vnn3 | chr10 | protein_coding | vanin 3 |
