# Supplementary material for: CPEB3 regulates neuron-specific alternative splicing and involves neurogenesis gene expression
Source: Aging (Albany NY). 2020 Dec 9;13(2):2330–47. doi: 10.18632/aging.202259 (PMC7880327; doi:10.18632/aging.202259)
Supplement: Supplementary Tables 3 and 4 [file aging-13-202259-s003.pdf]

SUPPLEMENTARY TABLES

Supplementary Table 3. Alternative splicing events.

Known AS events

#Sample	3pMXE	5pMXE	A3SS	A3SS&ES	A5SS	A5SS&ES	ES	IntronR	MXE	cassetteExon	Total	Detected junction	AS/100SJ
CPEB3_1st	416	1373	3565	323	4734	416	2019	2009	179	1158	16192	188308	8.6
CPEB3_2nd	388	1359	3420	296	4500	398	1949	1978	186	1032	15506	178425	8.69
CPEB3_3rd	440	1385	3634	293	4825	410	2074	1963	203	1191	16418	199214	8.24
Ctrl_1st	380	1291	3283	280	4443	376	1930	1979	165	1048	15175	178945	8.48
Ctrl_2nd	381	1276	3341	267	4542	384	1834	1883	170	1011	15089	172304	8.76
Ctrl_3rd	365	1119	3334	275	4109	361	1829	1890	171	966	14419	169493	8.51
Total	991	3102	8138	843	10127	972	3885	4632	406	2032	35128	311977	11.26

Novel AS events

Sample	3pMXE	5pMXE	A3SS	A3SS&ES	A5SS	A5SS&ES	ES	IntronR	MXE	cassetteExon	Total	Detected junction	AS/100SJ
Ctrl_1st	735	1859	4468	510	5566	632	2264	7407	273	959	24673	59714	41.32
Ctrl_2nd	728	1857	4313	488	5613	593	1962	6803	244	964	23565	57352	41.09
SRSF6_1st	796	1933	4765	548	6353	678	2085	7436	287	1115	25996	71257	36.48
SRSF6_2nd	724	1955	5178	585	6504	657	2361	6885	325	1188	26362	77410	34.06
Total	1982	4606	12609	1539	15249	1769	5379	15388	790	2684	61995	167859	36.93

All AS events

#Sample	3pMXE	5pMXE	A3SS	A3SS&ES	A5SS	A5SS&ES	ES	IntronR	MXE	cassetteExon	Total	Detected junction	AS/100SJ
Ctrl_1st	1037	2443	7283	821	8777	1040	4495	8522	546	2528	37492	200907	18.66
Ctrl_2nd	1040	2424	7194	815	8932	1032	4169	7955	520	2555	36636	199202	18.39
SRSF6_1st	1111	2549	7705	884	9876	1108	4408	8592	574	2767	39574	213797	18.51
SRSF6_2nd	1053	2573	8200	920	10070	1099	4778	8045	605	2871	40214	219556	18.32
Total	2461	5485	16857	2013	20314	2389	8571	17014	1269	5026	81399	326115	24.96

Supplementary Table 4. CPEB3-OE versus Ctrl differential ASs (RAS).

Sample	Type	3pMXE	5pMXE	A3SS	A3SS&ES	A5SS	A5SS&ES	ES	IntronR	MXE	cassetteExon	Total
CPEB3_vs_Ctrl	Up	4	13	60	2	71	3	39	48	6	22	268
CPEB3_vs_Ctrl	Down	9	13	44	2	65	3	33	34	5	21	229
