# Supplementary material for: CPEB3 regulates neuron-specific alternative splicing and involves neurogenesis gene expression
Source: Aging (Albany NY). 2020 Dec 9;13(2):2330–47. doi: 10.18632/aging.202259 (PMC7880327; doi:10.18632/aging.202259)
Supplement: Supplementary Table 5 [file aging-13-202259-s004.docx]

**Supplementary Table 5. GO enrichment and KEGG pathway of RAS.**

RAS_GO_terms

| **#Term** | **Database** | **ID** | **Input number** | **Background number** | **P-Value** | **Corrected P-Value** | **Input** | **Hyperlink** |
| --- | --- | --- | --- | --- | --- | --- | --- | --- |
| regulation of transcription, DNA-dependent | Gene Ontology | GO:0006355 | 56 | 1845 | 0.000219104 | 0.009762097 | Srsf5\|Sbno2\|Zfp280c\|Psip1\|Ralgapa1\|Nfat5\|Paxbp1\|Bach2\|Parp14\|Zmynd11\|Osr1\|Zfp1\|Pura\|Hnrnpk\|Crem\|Cipc\|Zbtb49\|Pknox2\|Arid3a\|Irf7\|Pmf1\|Sp1\|Smad6\|Jade1\|Nfkbiz\|Sap130\|Mbd3\|Tcf12\|Smarca2\|Meaf6\|Nr1h3\|Phtf1\|Sltm\|Nfe2l1\|Ubtf\|Rpap2\|Med24\|Men1\|Kdm4b\|Med7\|Tead1\|Rlim\|Zfp740\|Brd2\|Snip1\|Gtf2h2\|Tgif1\|Bahd1\|Sf1\|Srsf10\|Med16\|Tbp\|Wdr61\|Mier2\|Ncor1\|Foxn3 | http://amigo.geneontology.org/amigo/term/GO:0006355 |
| RNA splicing | Gene Ontology | GO:0008380 | 13 | 222 | 0.000320069 | 0.009762097 | Srsf5\|Srsf7\|Ppil3\|U2af1\|Arl6ip4\|Sf1\|U2af1l4\|Hnrnpk\|Srsf10\|Ppih\|Thoc2\|Ptbp1\|Hnrnpf | http://amigo.geneontology.org/amigo/term/GO:0008380 |
| transcription, DNA-dependent | Gene Ontology | GO:0006351 | 52 | 1759 | 0.000709254 | 0.014421505 | Srsf5\|Sbno2\|Zfp280c\|Psip1\|Nfat5\|Bach2\|Parp14\|Cipc\|Osr1\|Zfp1\|Pura\|Hnrnpk\|Crem\|Zmynd11\|Zbtb49\|Tgif1\|Arid3a\|Irf7\|Rlim\|Pmf1\|Tbp\|Mier2\|Zfp740\|Nfkbiz\|Gtf3c2\|Sap130\|Mbd3\|Tcf12\|Tead1\|Meaf6\|Nr1h3\|Phtf1\|Sltm\|Ubtf\|Rpap2\|Men1\|Med24\|Kdm4b\|Med7\|Paxbp1\|Smarca2\|Brd2\|Gtf2h2\|Bahd1\|Sf1\|Jade1\|Med16\|Sp1\|Wdr61\|Smad6\|Ncor1\|Foxn3 | http://amigo.geneontology.org/amigo/term/GO:0006351 |
| mRNA processing | Gene Ontology | GO:0006397 | 14 | 295 | 0.001516767 | 0.023130693 | Srsf5\|Srsf7\|U2af1\|Arl6ip4\|Sf1\|Hnrnpk\|U2af1l4\|Ppil3\|Srsf10\|Ppih\|Thoc2\|Ptbp1\|Papola\|Hnrnpf | http://amigo.geneontology.org/amigo/term/GO:0006397 |
| positive regulation of transcription from RNA polymerase II promoter | Gene Ontology | GO:0045944 | 26 | 758 | 0.0025208 | 0.027881678 | Sbno2\|Psip1\|Strn3\|Nfat5\|Osr1\|Hnrnpk\|Crem\|Arid3a\|Irf7\|Traf6\|Sp1\|Mllt10\|Tcf12\|Nlrc5\|Smarca2\|Nr1h3\|Nfe2l1\|Hax1\|Men1\|Paxbp1\|Tead1\|Hras\|Mtf2\|Senp1\|Wdr61\|Jade1 | http://amigo.geneontology.org/amigo/term/GO:0045944 |
| mRNA transport | Gene Ontology | GO:0051028 | 6 | 74 | 0.00274246 | 0.027881678 | Srsf7\|Myo1c\|G3bp2\|Thoc2\|Ranbp17\|Eif5a | http://amigo.geneontology.org/amigo/term/GO:0051028 |
| response to DNA damage stimulus | Gene Ontology | GO:0006974 | 15 | 369 | 0.004586349 | 0.039966755 | Recql5\|Zranb3\|Rassf1\|Gtf2h2\|Brcc3\|2310003H01Rik\|Aplf\|Ap5s1\|Macrod2\|Ikbke\|Bbc3\|Trip12\|Men1\|Dclre1c\|Fbxo31 | http://amigo.geneontology.org/amigo/term/GO:0006974 |
| cell division | Gene Ontology | GO:0051301 | 14 | 344 | 0.006013197 | 0.045850628 | Recql5\|Mapre3\|Pmf1\|Cdc27\|Tacc1\|Anapc16\|Sgol1\|Cdc25c\|Sept9\|Aurka\|Wapal\|Chfr\|Sept11\|Bora | http://amigo.geneontology.org/amigo/term/GO:0051301 |
| protein ubiquitination | Gene Ontology | GO:0016567 | 9 | 180 | 0.00734489 | 0.049782033 | Kctd13\|Smurf1\|Traf3\|Traf6\|Anapc16\|Wdsub1\|March2\|Chfr\|Rlim | http://amigo.geneontology.org/amigo/term/GO:0016567 |
| mitosis | Gene Ontology | GO:0007067 | 11 | 256 | 0.00968015 | 0.059048915 | Recql5\|Mapre3\|Pmf1\|Cdc25c\|Anapc16\|Sgol1\|Espl1\|Aurka\|Wapal\|Chfr\|Bora | http://amigo.geneontology.org/amigo/term/GO:0007067 |
| protein heterooligomerization | Gene Ontology | GO:0051291 | 5 | 77 | 0.015270819 | 0.081596114 | Sept9\|Hras\|Sept11\|Med24\|Prkab1 | http://amigo.geneontology.org/amigo/term/GO:0051291 |
| cell cycle | Gene Ontology | GO:0007049 | 19 | 590 | 0.017085342 | 0.081596114 | Recql5\|Cdc27\|Mapre3\|Pmf1\|Rassf1\|Tacc1\|Fbxo31\|Anapc16\|Sgol1\|Cdc25c\|Ccndbp1\|Sept9\|Aurka\|Wapal\|Pak4\|Chfr\|Sept11\|Bora\|Foxn3 | http://amigo.geneontology.org/amigo/term/GO:0007049 |
| regulation of translation | Gene Ontology | GO:0006417 | 6 | 109 | 0.017389336 | 0.081596114 | Mif4gd\|Eif4g2\|Mknk1\|Paip1\|Pum1\|Eif4ebp2 | http://amigo.geneontology.org/amigo/term/GO:0006417 |
| immune system process | Gene Ontology | GO:0002376 | 12 | 324 | 0.020741619 | 0.090374199 | Otud7b\|Irf7\|Traf6\|Tec\|Csf1\|Traf3\|Ecsit\|C8g\|Bag6\|Nlrc5\|Dclre1c\|Cadm1 | http://amigo.geneontology.org/amigo/term/GO:0002376 |
| endocytosis | Gene Ontology | GO:0006897 | 7 | 149 | 0.023271634 | 0.094637979 | Gapvd1\|Hras\|Fcho2\|Dnm1\|March2\|Dnm1l\|Ap2b1 | http://amigo.geneontology.org/amigo/term/GO:0006897 |
| chromatin modification | Gene Ontology | GO:0016568 | 10 | 259 | 0.025873348 | 0.09606532 | Bag6\|Meaf6\|Mtf2\|Brcc3\|Bahd1\|Men1\|Kdm4b\|Brd2\|Zmynd11\|Ncor1 | http://amigo.geneontology.org/amigo/term/GO:0016568 |
| intracellular protein transport | Gene Ontology | GO:0006886 | 8 | 188 | 0.026772302 | 0.09606532 | Aspscr1\|Tnpo1\|Sec23b\|Snx10\|Ap4b1\|Ap2b1\|Ranbp17\|Snapin | http://amigo.geneontology.org/amigo/term/GO:0006886 |
| protein dephosphorylation | Gene Ontology | GO:0006470 | 6 | 122 | 0.028410917 | 0.09628144 | Ptrhd1\|Ppm1e\|Cdc25c\|Ssh2\|Ssh3\|Dusp14 | http://amigo.geneontology.org/amigo/term/GO:0006470 |
| protein autophosphorylation | Gene Ontology | GO:0046777 | 7 | 159 | 0.031647465 | 0.101605019 | Ulk3\|Ptk2\|Ephb4\|Wnk1\|Mark2\|Clk2\|Pak2 | http://amigo.geneontology.org/amigo/term/GO:0046777 |
| peptidyl-tyrosine phosphorylation | Gene Ontology | GO:0018108 | 5 | 98 | 0.03828692 | 0.116775107 | Abi1\|Clk2\|Ptk2\|Ephb4\|Tec | http://amigo.geneontology.org/amigo/term/GO:0018108 |
| DNA repair | Gene Ontology | GO:0006281 | 10 | 290 | 0.049569392 | 0.143987283 | Recql5\|Zranb3\|Gtf2h2\|Polg2\|Brcc3\|2310003H01Rik\|Aplf\|Ap5s1\|Trip12\|Dclre1c | http://amigo.geneontology.org/amigo/term/GO:0006281 |
| cell proliferation | Gene Ontology | GO:0008283 | 7 | 180 | 0.055275674 | 0.153264367 | Ogfod1\|Tacc1\|Hras\|Bad\|Csf1\|Scrib\|Pura | http://amigo.geneontology.org/amigo/term/GO:0008283 |
| protein transport | Gene Ontology | GO:0015031 | 16 | 557 | 0.064363347 | 0.17070279 | Fam160a2\|Tnpo1\|C2cd5\|Sec23b\|Snx10\|Myo1c\|Sys1\|Ap5s1\|Stxbp2\|Timm10b\|Mcfd2\|Trim3\|Ap4b1\|Ap2b1\|Ranbp17\|Eif5a | http://amigo.geneontology.org/amigo/term/GO:0015031 |
| regulation of apoptotic process | Gene Ontology | GO:0042981 | 5 | 116 | 0.069210717 | 0.175910573 | Bad\|Traf3\|Bag6\|Pak2\|Traf6 | http://amigo.geneontology.org/amigo/term/GO:0042981 |
| transcription from RNA polymerase II promoter | Gene Ontology | GO:0006366 | 12 | 398 | 0.076185585 | 0.18588447 | Irf7\|Gtf2h2\|Tbp\|Nfe2l1\|Med24\|Hnrnpk\|Med16\|Sp1\|Psip1\|Smarca2\|Jade1\|Nr1h3 | http://amigo.geneontology.org/amigo/term/GO:0006366 |
| positive regulation of gene expression | Gene Ontology | GO:0010628 | 7 | 197 | 0.080808424 | 0.18588447 | Nfat5\|Prkab1\|Cd44\|Hras\|Csf1\|Osr1\|Tcf12 | http://amigo.geneontology.org/amigo/term/GO:0010628 |
| translation | Gene Ontology | GO:0006412 | 8 | 240 | 0.086193639 | 0.18588447 | Mrpl24\|Eif4g2\|Eif2d\|Wars\|Rps28\|Mrps17\|Eftud1\|Eif5a | http://amigo.geneontology.org/amigo/term/GO:0006412 |
| DNA replication | Gene Ontology | GO:0006260 | 5 | 124 | 0.086352955 | 0.18588447 | Kctd13\|Recql5\|Polg2\|Nol8\|Repin1 | http://amigo.geneontology.org/amigo/term/GO:0006260 |
| protein phosphorylation | Gene Ontology | GO:0006468 | 16 | 584 | 0.088838877 | 0.18588447 | Ulk3\|Ptk2\|Tec\|Ephb4\|Prkab1\|Gtf2h2\|Wnk1\|Map2k2\|Aurka\|Mark2\|Ikbke\|Pak4\|Mknk1\|Clk2\|Mapkapk3\|Pak2 | http://amigo.geneontology.org/amigo/term/GO:0006468 |
| dephosphorylation | Gene Ontology | GO:0016311 | 6 | 164 | 0.091418592 | 0.18588447 | Ppap2c\|Nt5m\|Inpp4a\|Ssh2\|Ssh3\|Dusp14 | http://amigo.geneontology.org/amigo/term/GO:0016311 |

RAS_KEGG_pathway

| **#Term** | **Database** | **ID** | **Input number** | **Background**  **number** | **P-Value** | **Corrected P-Value** | **Input** | **Hyperlink** |
| --- | --- | --- | --- | --- | --- | --- | --- | --- |
| Spliceosome | KEGG PATHWAY | mmu03040 | 8 | 132 | 0.001574191 | 0.248722212 | Srsf5\|Srsf7\|U2af1\|U2af1l4\|Hnrnpk\|Srsf10\|Ppih\|Thoc2 | http://www.genome.jp/kegg-bin/show_pathway?mmu03040/mmu:108121%09red/mmu:331401%09red/mmu:15387%09red/mmu:20384%09red/mmu:225027%09red/mmu:66101%09red/mmu:14105%09red/mmu:233073%09red |
| RIG-I-like receptor signaling pathway | KEGG PATHWAY | mmu04622 | 5 | 69 | 0.00573197 | 0.364549536 | Irf7\|Traf3\|Cyld\|Traf6\|Ikbke | http://www.genome.jp/kegg-bin/show_pathway?mmu04622/mmu:74256%09red/mmu:22031%09red/mmu:56489%09red/mmu:22034%09red/mmu:54123%09red |
| Herpes simplex infection | KEGG PATHWAY | mmu05168 | 9 | 204 | 0.006934073 | 0.364549536 | Srsf5\|Srsf7\|Traf3\|Irf7\|Traf6\|Tbp\|H2-T24\|Hnrnpk\|Ikbke | http://www.genome.jp/kegg-bin/show_pathway?mmu05168/mmu:22031%09red/mmu:15387%09red/mmu:22034%09red/mmu:225027%09red/mmu:15042%09red/mmu:54123%09red/mmu:56489%09red/mmu:20384%09red/mmu:21374%09red |
| Viral carcinogenesis | KEGG PATHWAY | mmu05203 | 9 | 228 | 0.013792457 | 0.364549536 | Traf3\|Irf7\|Gtf2h2\|H2-T24\|Scrib\|Bad\|Hnrnpk\|Tbp\|Vac14 | http://www.genome.jp/kegg-bin/show_pathway?mmu05203/mmu:22031%09red/mmu:15387%09red/mmu:23894%09red/mmu:21374%09red/mmu:15042%09red/mmu:54123%09red/mmu:12015%09red/mmu:234729%09red/mmu:105782%09red |
| ErbB signaling pathway | KEGG PATHWAY | mmu04012 | 5 | 87 | 0.014877273 | 0.364549536 | Pak4\|Map2k2\|Bad\|Ptk2\|Pak2 | http://www.genome.jp/kegg-bin/show_pathway?mmu04012/mmu:224105%09red/mmu:12015%09red/mmu:70584%09red/mmu:14083%09red/mmu:26396%09red |
| VEGF signaling pathway | KEGG PATHWAY | mmu04370 | 4 | 62 | 0.019607083 | 0.364549536 | Map2k2\|Bad\|Ptk2\|Mapkapk3 | http://www.genome.jp/kegg-bin/show_pathway?mmu04370/mmu:102626%09red/mmu:26396%09red/mmu:14083%09red/mmu:12015%09red |
| Hepatitis C | KEGG PATHWAY | mmu05160 | 6 | 136 | 0.025952412 | 0.364549536 | Traf3\|Irf7\|Traf6\|Bad\|Ikbke\|Nr1h3 | http://www.genome.jp/kegg-bin/show_pathway?mmu05160/mmu:22259%09red/mmu:22031%09red/mmu:22034%09red/mmu:54123%09red/mmu:56489%09red/mmu:12015%09red |
| Toll-like receptor signaling pathway | KEGG PATHWAY | mmu04620 | 5 | 101 | 0.026565741 | 0.364549536 | Map2k2\|Traf3\|Irf7\|Traf6\|Ikbke | http://www.genome.jp/kegg-bin/show_pathway?mmu04620/mmu:22031%09red/mmu:54123%09red/mmu:56489%09red/mmu:22034%09red/mmu:26396%09red |
| Bladder cancer | KEGG PATHWAY | mmu05219 | 3 | 39 | 0.026817017 | 0.364549536 | Rassf1\|Map2k2\|Mmp2 | http://www.genome.jp/kegg-bin/show_pathway?mmu05219/mmu:17390%09red/mmu:56289%09red/mmu:26396%09red |
| Ubiquitin mediated proteolysis | KEGG PATHWAY | mmu04120 | 6 | 141 | 0.030279257 | 0.364549536 | Smurf1\|Ube4a\|Traf6\|Cdc27\|Uba7\|Trip12 | http://www.genome.jp/kegg-bin/show_pathway?mmu04120/mmu:14897%09red/mmu:217232%09red/mmu:75788%09red/mmu:22034%09red/mmu:74153%09red/mmu:140630%09red |
| Endocytosis | KEGG PATHWAY | mmu04144 | 8 | 220 | 0.030511744 | 0.364549536 | Smurf1\|Traf6\|Smad6\|H2-T24\|Dnm1\|Git2\|Iqsec2\|Ap2b1 | http://www.genome.jp/kegg-bin/show_pathway?mmu04144/mmu:26431%09red/mmu:75788%09red/mmu:22034%09red/mmu:245666%09red/mmu:13429%09red/mmu:15042%09red/mmu:17130%09red/mmu:71770%09red |
| Ether lipid metabolism | KEGG PATHWAY | mmu00565 | 3 | 41 | 0.030541315 | 0.364549536 | Pla2g2e\|Ppap2c\|Ept1 | http://www.genome.jp/kegg-bin/show_pathway?mmu00565/mmu:26970%09red/mmu:28042%09red/mmu:50784%09red |
| Transcriptional misregulation in cancer | KEGG PATHWAY | mmu05202 | 7 | 180 | 0.030674244 | 0.364549536 | Aspscr1\|Ptk2\|Sp1\|Golph3l\|Men1\|Nfkbiz\|Ncor1 | http://www.genome.jp/kegg-bin/show_pathway?mmu05202/mmu:229593%09red/mmu:80859%09red/mmu:17283%09red/mmu:14083%09red/mmu:20683%09red/mmu:68938%09red/mmu:20185%09red |
| Huntington's disease | KEGG PATHWAY | mmu05016 | 7 | 182 | 0.032301858 | 0.364549536 | Atp5c1\|Sp1\|Ndufs5\|Tbp\|Bbc3\|Ap2b1\|Dctn4 | http://www.genome.jp/kegg-bin/show_pathway?mmu05016/mmu:595136%09red/mmu:170770%09red/mmu:67665%09red/mmu:21374%09red/mmu:11949%09red/mmu:20683%09red/mmu:71770%09red |
| Bacterial invasion of epithelial cells | KEGG PATHWAY | mmu05100 | 4 | 77 | 0.039377071 | 0.410721484 | Dnm1\|Ptk2\|Sept11\|Sept9 | http://www.genome.jp/kegg-bin/show_pathway?mmu05100/mmu:53860%09red/mmu:13429%09red/mmu:14083%09red/mmu:52398%09red |
| Oocyte meiosis | KEGG PATHWAY | mmu04114 | 5 | 114 | 0.041592049 | 0.410721484 | Cdc25c\|Cdc27\|Espl1\|Aurka\|Sgol1 | http://www.genome.jp/kegg-bin/show_pathway?mmu04114/mmu:217232%09red/mmu:20878%09red/mmu:105988%09red/mmu:12532%09red/mmu:72415%09red |
| Non-small cell lung cancer | KEGG PATHWAY | mmu05223 | 3 | 55 | 0.063656284 | 0.563168546 | Rassf1\|Map2k2\|Bad | http://www.genome.jp/kegg-bin/show_pathway?mmu05223/mmu:56289%09red/mmu:12015%09red/mmu:26396%09red |
| Glycerophospholipid metabolism | KEGG PATHWAY | mmu00564 | 4 | 91 | 0.065296868 | 0.563168546 | Pla2g2e\|Agpat3\|Ppap2c\|Ept1 | http://www.genome.jp/kegg-bin/show_pathway?mmu00564/mmu:28169%09red/mmu:26970%09red/mmu:28042%09red/mmu:50784%09red |
| Axon guidance | KEGG PATHWAY | mmu04360 | 5 | 131 | 0.0677228 | 0.563168546 | Pak4\|Ptk2\|Ngef\|Ephb4\|Pak2 | http://www.genome.jp/kegg-bin/show_pathway?mmu04360/mmu:224105%09red/mmu:53972%09red/mmu:70584%09red/mmu:14083%09red/mmu:13846%09red |
| Regulation of actin cytoskeleton | KEGG PATHWAY | mmu04810 | 7 | 218 | 0.071896512 | 0.567982447 | Ptk2\|Map2k2\|Ssh2\|Ssh3\|Pak4\|Baiap2\|Pak2 | http://www.genome.jp/kegg-bin/show_pathway?mmu04810/mmu:224105%09red/mmu:26396%09red/mmu:14083%09red/mmu:245857%09red/mmu:237860%09red/mmu:108100%09red/mmu:70584%09red |
| mRNA surveillance pathway | KEGG PATHWAY | mmu03015 | 4 | 97 | 0.078578035 | 0.580578793 | Pcf11\|Msi1\|Pabpc4\|Papola | http://www.genome.jp/kegg-bin/show_pathway?mmu03015/mmu:17690%09red/mmu:74737%09red/mmu:230721%09red/mmu:18789%09red |
| Thyroid cancer | KEGG PATHWAY | mmu05216 | 2 | 29 | 0.083800753 | 0.580578793 | Map2k2\|Ncoa4 | http://www.genome.jp/kegg-bin/show_pathway?mmu05216/mmu:26396%09red/mmu:27057%09red |
| Synaptic vesicle cycle | KEGG PATHWAY | mmu04721 | 3 | 62 | 0.084514634 | 0.580578793 | Tcirg1\|Dnm1\|Ap2b1 | http://www.genome.jp/kegg-bin/show_pathway?mmu04721/mmu:27060%09red/mmu:71770%09red/mmu:13429%09red |
| Nicotinate and nicotinamide metabolism | KEGG PATHWAY | mmu00760 | 2 | 31 | 0.093963925 | 0.618595839 | Nmnat3\|Nt5m | http://www.genome.jp/kegg-bin/show_pathway?mmu00760/mmu:103850%09red/mmu:74080%09red |
| Renal cell carcinoma | KEGG PATHWAY | mmu05211 | 3 | 68 | 0.104406397 | 0.644840961 | Pak4\|Map2k2\|Pak2 | http://www.genome.jp/kegg-bin/show_pathway?mmu05211/mmu:224105%09red/mmu:26396%09red/mmu:70584%09red |
| T cell receptor signaling pathway | KEGG PATHWAY | mmu04660 | 4 | 108 | 0.106113069 | 0.644840961 | Pak4\|Map2k2\|Tec\|Pak2 | http://www.genome.jp/kegg-bin/show_pathway?mmu04660/mmu:224105%09red/mmu:26396%09red/mmu:70584%09red/mmu:21682%09red |
| Prion diseases | KEGG PATHWAY | mmu05020 | 2 | 35 | 0.115259733 | 0.674482885 | Map2k2\|C8g | http://www.genome.jp/kegg-bin/show_pathway?mmu05020/mmu:26396%09red/mmu:69379%09red |
| RNA degradation | KEGG PATHWAY | mmu03018 | 3 | 77 | 0.137277508 | 0.702469863 | Ddx6\|Wdr61\|Pabpc4 | http://www.genome.jp/kegg-bin/show_pathway?mmu03018/mmu:13209%09red/mmu:230721%09red/mmu:66317%09red |
| MAPK signaling pathway | KEGG PATHWAY | mmu04010 | 7 | 258 | 0.139691016 | 0.702469863 | Traf6\|Mknk1\|Map2k2\|Ecsit\|Dusp14\|Mapkapk3\|Pak2 | http://www.genome.jp/kegg-bin/show_pathway?mmu04010/mmu:224105%09red/mmu:26396%09red/mmu:56405%09red/mmu:22034%09red/mmu:26940%09red/mmu:102626%09red/mmu:17346%09red |
