# Supplementary material for: CPEB3 regulates neuron-specific alternative splicing and involves neurogenesis gene expression
Source: Aging (Albany NY). 2020 Dec 9;13(2):2330–47. doi: 10.18632/aging.202259 (PMC7880327; doi:10.18632/aging.202259)
Supplement: Supplementary Table 6 [file aging-13-202259-s005.pdf]

SUPPLEMENTARY TABLES

Supplementary Table 6. DEG_Primer Sequence

Gene	Sequence
DEG-Cpeb3-F	GCTGATTGGAGAACGCAACA
DEG-Cpeb3-R	AACTGGCAATGATAATGGACCG
DEG-Saa3-F	TGATGACTTTAGCAGCCCAGG
DEG-Saa3-R	CGAGCCTACTCTGACATGAAGA
DEG-Lcn2-F	TTTCAGAAGTCTTTCGGAAA
DEG-Lcn2-R	CTCAGGTACAGAGCTACAA

AS_Primer Sequence

Gene	Sequence
Traf6-M/A-F	AAGCCAGAAGAAGGTGTCAGA
Traf6-M-R	GTCAGTCGATCTGCTCAGGA
Traf6-A-R	GCCTTCCTATCTGCTCAGGA
Cyld-M/A-R	GGCCTGAACTCATTGTGACAGT
Cyld-M-F	CGCGCCTGAGGCACTTTGAA
Cyld-A-F	GTGGCTCCTGGCACTTTGAA
Nrp2-M/A-R	TGATGGAGTGATCGGTCTTCT
Nrp2-M-F	GATTTTAAAGGGGGCACCCT
Nrp2-A-F	GCTTTTGCAGGGGGCACCCT
Nav2-M/A-R	GGTGAGGAAGGCAATGAAGAAT
Nav2-M-F	GTCAATGAGTTGCGCAGCT
Nav2-A-F	GAAAAACTGGTTGCGCAGCT
Jade1-M/A-R	TGTCGTCAGAATCCTCACTGCT
Jade1-M-F	TGCGCAGCAGGCTGCCTG
Jade1- A-F	CCTCCCACCGGCTGCCTG
Naa16-M/A-R	TAGACGAGAGGCTTCAGCTT
Naa16-M-F	AACTTCTTACCTGGGGCGAA
Naa16-A-F	TTATTGAAACCTGGGGCGAA
Dnm1l-M/A-F	GCTGGCATAATTGGAATTGGTT
Dnm1l-M-R	TGATGGCAAGGTCCCATCTG
Dnm1l-A-R	ACGGGACAAGGTCCCATCTG
H2-T24-M/A-R	CCTGACCTGGCAGTTGAATG
H2-T24-M-F	TGAAGAGGCTCCCATTTCAG
H2-T24-A-F	TTCTCCTTACCCCATTTCAG

RIP_Primer Sequence

Gene	Sequence
Cyld-F	ACGTGGGCAGTCCTGTGAA
Cyld-R	GACACCGTCCTCTCCGCTAA
Nav2-1-F	ACTCCCAGGTCCTACCGCAA
Nav2-1-R	GGGTGGAGAGGTGACAGGTT
Nav2-2-F	CTCTGTCACCACCGCCGTG

Nav2-2-R	CTCGAACACCAAGCACCTGAG
Nrp2-F	ACTTCACAACCCAAGGCTCTG
Nrp2-R	AAACAACAGGGCCTGTCTCT
Lcn2-F	GTCTTAACCGCTGAGCCATC
Lcn2-R	CACCTGTCTTAACACCACCAAT
Baiap2-F	ACAGTGTGAGGACGCTGAC
Baiap2-R	AACAACCCAAGAACAAACCAAC
Dnm1l-F	TTGTCCCGTGATCCAGCT
Dnm1l-R	GAACAAAGAAGAAACAGGCTAG
Naa16-F	GGAGCAGGTCTCACTATGTAGC
Naa16-R	CTTGAAAGGCTGAGGCAGAAGA
Jade1-1-F	TTGTTCCTGTCGCCTCGG
Jade1-1-R	GGGAAACAGGCTGGAGACC
Jade1-2-F	GTGGGCAGGTGCTGGTTG
Jade1-2-R	CGGGCAGCGACAAGGAAC
