# Supplementary material for: CPEB3 regulates neuron-specific alternative splicing and involves neurogenesis gene expression
Source: Aging (Albany NY). 2020 Dec 9;13(2):2330–47. doi: 10.18632/aging.202259 (PMC7880327; doi:10.18632/aging.202259)
Supplement: Supplementary Table 7 [file aging-13-202259-s006.docx]

**Supplementary Table 7. GO enrichment and KEGG pathway of CPEB3 binding genes.**

| **#Term** | **Database** | **ID** | **Input number** | **Background number** | **P-Value** | **Corrected P-Value** | **Input** | **Hyperlink** |
| --- | --- | --- | --- | --- | --- | --- | --- | --- |
| peroxisome fission | Gene Ontology | GO:0016559 | 2 | 9 | 0.000829652 | 0.102198709 | Dnm1l\|Acot8 | http://amigo.geneontology.org/amigo/term/GO:0016559 |
| positive regulation of release of cytochrome c from mitochondria | Gene Ontology | GO:0090200 | 2 | 21 | 0.004660026 | 0.102198709 | Dnm1l\|Bbc3 | http://amigo.geneontology.org/amigo/term/GO:0090200 |
| dUMP catabolic process | Gene Ontology | GO:0046079 | 1 | 1 | 0.004892218 | 0.102198709 | Nt5m | http://amigo.geneontology.org/amigo/term/GO:0046079 |
| random inactivation of X chromosome | Gene Ontology | GO:0060816 | 1 | 1 | 0.004892218 | 0.102198709 | Rlim | http://amigo.geneontology.org/amigo/term/GO:0060816 |
| cellular chemical homeostasis | Gene Ontology | GO:0055082 | 1 | 1 | 0.004892218 | 0.102198709 | Wdtc1 | http://amigo.geneontology.org/amigo/term/GO:0055082 |
| synaptic vesicle fusion to presynaptic membrane | Gene Ontology | GO:0031629 | 1 | 1 | 0.004892218 | 0.102198709 | Snapin | http://amigo.geneontology.org/amigo/term/GO:0031629 |
| dynamin polymerization involved in mitochondrial fission | Gene Ontology | GO:0003374 | 1 | 1 | 0.004892218 | 0.102198709 | Dnm1l | http://amigo.geneontology.org/amigo/term/GO:0003374 |
| membrane fission involved in mitochondrial fission | Gene Ontology | GO:0090149 | 1 | 1 | 0.004892218 | 0.102198709 | Dnm1l | http://amigo.geneontology.org/amigo/term/GO:0090149 |
| polyphosphate catabolic process | Gene Ontology | GO:0006798 | 1 | 1 | 0.004892218 | 0.102198709 | Inpp4a | http://amigo.geneontology.org/amigo/term/GO:0006798 |
| GO term Description | Gene Ontology | GO term | 1 | 1 | 0.004892218 | 0.102198709 | Gene | http://amigo.geneontology.org/amigo/term/GO term |
| regulation of calcineurin-NFAT signaling cascade | Gene Ontology | GO:0070884 | 1 | 1 | 0.004892218 | 0.102198709 | Nfat5 | http://amigo.geneontology.org/amigo/term/GO:0070884 |
| macrophage fusion | Gene Ontology | GO:0034238 | 1 | 1 | 0.004892218 | 0.102198709 | Cd44 | http://amigo.geneontology.org/amigo/term/GO:0034238 |
| release of cytochrome c from mitochondria | Gene Ontology | GO:0001836 | 2 | 22 | 0.005109935 | 0.102198709 | Dnm1l\|Bbc3 | http://amigo.geneontology.org/amigo/term/GO:0001836 |
| positive regulation of intrinsic apoptotic signaling pathway | Gene Ontology | GO:2001244 | 2 | 28 | 0.008205623 | 0.110340326 | Dnm1l\|Bbc3 | http://amigo.geneontology.org/amigo/term/GO:2001244 |
| regulation of protein oligomerization | Gene Ontology | GO:0032459 | 1 | 2 | 0.009760875 | 0.110340326 | Dnm1l | http://amigo.geneontology.org/amigo/term/GO:0032459 |
| regulation of lipid transport by positive regulation of transcription from RNA polymerase II promoter | Gene Ontology | GO:0072369 | 1 | 2 | 0.009760875 | 0.110340326 | Hnrnpk | http://amigo.geneontology.org/amigo/term/GO:0072369 |
| positive regulation of monocyte aggregation | Gene Ontology | GO:1900625 | 1 | 2 | 0.009760875 | 0.110340326 | Cd44 | http://amigo.geneontology.org/amigo/term/GO:1900625 |
| positive regulation of translational termination | Gene Ontology | GO:0045905 | 1 | 2 | 0.009760875 | 0.110340326 | Eif5a | http://amigo.geneontology.org/amigo/term/GO:0045905 |
| regulation of low-density lipoprotein particle clearance | Gene Ontology | GO:0010988 | 1 | 2 | 0.009760875 | 0.110340326 | Hnrnpk | http://amigo.geneontology.org/amigo/term/GO:0010988 |
| positive regulation of low-density lipoprotein particle receptor biosynthetic process | Gene Ontology | GO:0045716 | 1 | 2 | 0.009760875 | 0.110340326 | Hnrnpk | http://amigo.geneontology.org/amigo/term/GO:0045716 |
| dicarboxylic acid catabolic process | Gene Ontology | GO:0043649 | 1 | 2 | 0.009760875 | 0.110340326 | Acot8 | http://amigo.geneontology.org/amigo/term/GO:0043649 |
| regulation of peroxisome organization | Gene Ontology | GO:1900063 | 1 | 2 | 0.009760875 | 0.110340326 | Dnm1l | http://amigo.geneontology.org/amigo/term/GO:1900063 |
| translational frameshifting | Gene Ontology | GO:0006452 | 1 | 2 | 0.009760875 | 0.110340326 | Eif5a | http://amigo.geneontology.org/amigo/term/GO:0006452 |
| regulation of transcription, DNA-dependent | Gene Ontology | GO:0006355 | 16 | 1845 | 0.014258943 | 0.111693566 | Nfat5\|Phtf1\|Pmf1\|Snip1\|Gtf2h2\|Jade1\|Zfp280c\|Sf1\|Kdm4b\|Pura\|Hnrnpk\|Med7\|Tead1\|Rlim\|Pknox2\|Foxn3 | http://amigo.geneontology.org/amigo/term/GO:0006355 |
| regulation of mitochondrion organization | Gene Ontology | GO:0010821 | 1 | 3 | 0.014606082 | 0.111693566 | Dnm1l | http://amigo.geneontology.org/amigo/term/GO:0010821 |
| positive regulation of translational elongation | Gene Ontology | GO:0045901 | 1 | 3 | 0.014606082 | 0.111693566 | Eif5a | http://amigo.geneontology.org/amigo/term/GO:0045901 |
| production of miRNAs involved in gene silencing by miRNA | Gene Ontology | GO:0035196 | 1 | 3 | 0.014606082 | 0.111693566 | Snip1 | http://amigo.geneontology.org/amigo/term/GO:0035196 |
| positive regulation of protein insertion into mitochondrial membrane involved in apoptotic signaling pathway | Gene Ontology | GO:1900740 | 1 | 3 | 0.014606082 | 0.111693566 | Bbc3 | http://amigo.geneontology.org/amigo/term/GO:1900740 |
| protein localization in mitochondrion | Gene Ontology | GO:0070585 | 1 | 3 | 0.014606082 | 0.111693566 | Dnm1l | http://amigo.geneontology.org/amigo/term/GO:0070585 |
| wound healing involved in inflammatory response | Gene Ontology | GO:0002246 | 1 | 3 | 0.014606082 | 0.111693566 | Cd44 | http://amigo.geneontology.org/amigo/term/GO:0002246 |
| monocyte aggregation | Gene Ontology | GO:0070487 | 1 | 3 | 0.014606082 | 0.111693566 | Cd44 | http://amigo.geneontology.org/amigo/term/GO:0070487 |
| terminal button organization | Gene Ontology | GO:0072553 | 1 | 3 | 0.014606082 | 0.111693566 | Snapin | http://amigo.geneontology.org/amigo/term/GO:0072553 |
| fatty acid elongation | Gene Ontology | GO:0030497 | 1 | 3 | 0.014606082 | 0.111693566 | Tecr | http://amigo.geneontology.org/amigo/term/GO:0030497 |
| positive regulation of neutrophil apoptotic process | Gene Ontology | GO:0033031 | 1 | 3 | 0.014606082 | 0.111693566 | Cd44 | http://amigo.geneontology.org/amigo/term/GO:0033031 |
| regulation of early endosome to late endosome transport | Gene Ontology | GO:2000641 | 1 | 4 | 0.01942795 | 0.123201632 | Map2k2 | http://amigo.geneontology.org/amigo/term/GO:2000641 |
| peptidyl-lysine modification to hypusine | Gene Ontology | GO:0008612 | 1 | 4 | 0.01942795 | 0.123201632 | Eif5a | http://amigo.geneontology.org/amigo/term/GO:0008612 |
| lateral mesoderm development | Gene Ontology | GO:0048368 | 1 | 4 | 0.01942795 | 0.123201632 | Tead1 | http://amigo.geneontology.org/amigo/term/GO:0048368 |
| regulation of Golgi inheritance | Gene Ontology | GO:0090170 | 1 | 4 | 0.01942795 | 0.123201632 | Map2k2 | http://amigo.geneontology.org/amigo/term/GO:0090170 |
| cell-substrate junction assembly | Gene Ontology | GO:0007044 | 1 | 4 | 0.01942795 | 0.123201632 | Tln1 | http://amigo.geneontology.org/amigo/term/GO:0007044 |
| recognition of apoptotic cell | Gene Ontology | GO:0043654 | 1 | 4 | 0.01942795 | 0.123201632 | Pear1 | http://amigo.geneontology.org/amigo/term/GO:0043654 |
| DNA damage response, detection of DNA damage | Gene Ontology | GO:0042769 | 1 | 4 | 0.01942795 | 0.123201632 | Dnaja1 | http://amigo.geneontology.org/amigo/term/GO:0042769 |
| transcription, DNA-dependent | Gene Ontology | GO:0006351 | 15 | 1759 | 0.020535335 | 0.127123501 | Nfat5\|Zfp280c\|Pmf1\|Kdm4b\|Gtf2h2\|Phtf1\|Sf1\|Gtf3c2\|Pura\|Hnrnpk\|Med7\|Tead1\|Rlim\|Jade1\|Foxn3 | http://amigo.geneontology.org/amigo/term/GO:0006351 |
| negative regulation of translation | Gene Ontology | GO:0017148 | 2 | 48 | 0.023002502 | 0.136932897 | Pura\|Eif4ebp2 | http://amigo.geneontology.org/amigo/term/GO:0017148 |
| positive regulation of muscle cell differentiation | Gene Ontology | GO:0051149 | 1 | 5 | 0.024226589 | 0.136932897 | Eif5a | http://amigo.geneontology.org/amigo/term/GO:0051149 |
| positive regulation of cysteine-type endopeptidase activity | Gene Ontology | GO:2001056 | 1 | 5 | 0.024226589 | 0.136932897 | Bbc3 | http://amigo.geneontology.org/amigo/term/GO:2001056 |
| negative regulation of mature B cell apoptotic process | Gene Ontology | GO:0002906 | 1 | 5 | 0.024226589 | 0.136932897 | Cd44 | http://amigo.geneontology.org/amigo/term/GO:0002906 |
| reduction of endoplasmic reticulum calcium ion concentration | Gene Ontology | GO:0032471 | 1 | 6 | 0.029002111 | 0.140928198 | Bbc3 | http://amigo.geneontology.org/amigo/term/GO:0032471 |
| transcription from RNA polymerase III promoter | Gene Ontology | GO:0006383 | 1 | 6 | 0.029002111 | 0.140928198 | Gtf3c2 | http://amigo.geneontology.org/amigo/term/GO:0006383 |
| synaptic vesicle maturation | Gene Ontology | GO:0016188 | 1 | 6 | 0.029002111 | 0.140928198 | Snapin | http://amigo.geneontology.org/amigo/term/GO:0016188 |
| positive regulation of thymocyte apoptotic process | Gene Ontology | GO:0070245 | 1 | 6 | 0.029002111 | 0.140928198 | Bbc3 | http://amigo.geneontology.org/amigo/term/GO:0070245 |
| NIK/NF-kappaB cascade | Gene Ontology | GO:0038061 | 1 | 6 | 0.029002111 | 0.140928198 | Ikbke | http://amigo.geneontology.org/amigo/term/GO:0038061 |
| actin crosslink formation | Gene Ontology | GO:0051764 | 1 | 6 | 0.029002111 | 0.140928198 | Baiap2 | http://amigo.geneontology.org/amigo/term/GO:0051764 |
| N-terminal protein amino acid acetylation | Gene Ontology | GO:0006474 | 1 | 6 | 0.029002111 | 0.140928198 | Naa16 | http://amigo.geneontology.org/amigo/term/GO:0006474 |
| branching involved in prostate gland morphogenesis | Gene Ontology | GO:0060442 | 1 | 7 | 0.033754625 | 0.140928198 | Cd44 | http://amigo.geneontology.org/amigo/term/GO:0060442 |
| positive regulation of adaptive immune response | Gene Ontology | GO:0002821 | 1 | 7 | 0.033754625 | 0.140928198 | Cd44 | http://amigo.geneontology.org/amigo/term/GO:0002821 |
| very long-chain fatty acid biosynthetic process | Gene Ontology | GO:0042761 | 1 | 7 | 0.033754625 | 0.140928198 | Tecr | http://amigo.geneontology.org/amigo/term/GO:0042761 |
| ADP biosynthetic process | Gene Ontology | GO:0006172 | 1 | 7 | 0.033754625 | 0.140928198 | Atp5c1 | http://amigo.geneontology.org/amigo/term/GO:0006172 |
| negative regulation of fatty acid biosynthetic process | Gene Ontology | GO:0045717 | 1 | 7 | 0.033754625 | 0.140928198 | Wdtc1 | http://amigo.geneontology.org/amigo/term/GO:0045717 |
| DNA unwinding involved in replication | Gene Ontology | GO:0006268 | 1 | 7 | 0.033754625 | 0.140928198 | Pura | http://amigo.geneontology.org/amigo/term/GO:0006268 |
| hyaluronan catabolic process | Gene Ontology | GO:0030214 | 1 | 7 | 0.033754625 | 0.140928198 | Cd44 | http://amigo.geneontology.org/amigo/term/GO:0030214 |
| positive regulation of cell motility | Gene Ontology | GO:2000147 | 1 | 8 | 0.038484239 | 0.140928198 | Map2k2 | http://amigo.geneontology.org/amigo/term/GO:2000147 |
| negative regulation of retinoic acid receptor signaling pathway | Gene Ontology | GO:0048387 | 1 | 8 | 0.038484239 | 0.140928198 | Dhrs3 | http://amigo.geneontology.org/amigo/term/GO:0048387 |
| positive regulation of protein homooligomerization | Gene Ontology | GO:0032464 | 1 | 8 | 0.038484239 | 0.140928198 | Bbc3 | http://amigo.geneontology.org/amigo/term/GO:0032464 |
| positive regulation of heterotypic cell-cell adhesion | Gene Ontology | GO:0034116 | 1 | 8 | 0.038484239 | 0.140928198 | Cd44 | http://amigo.geneontology.org/amigo/term/GO:0034116 |
| notochord development | Gene Ontology | GO:0030903 | 1 | 8 | 0.038484239 | 0.140928198 | Tead1 | http://amigo.geneontology.org/amigo/term/GO:0030903 |
| histone H4-K12 acetylation | Gene Ontology | GO:0043983 | 1 | 8 | 0.038484239 | 0.140928198 | Jade1 | http://amigo.geneontology.org/amigo/term/GO:0043983 |
| regulation of steroid biosynthetic process | Gene Ontology | GO:0050810 | 1 | 8 | 0.038484239 | 0.140928198 | Sf1 | http://amigo.geneontology.org/amigo/term/GO:0050810 |
| regulation of stress-activated MAPK cascade | Gene Ontology | GO:0032872 | 1 | 8 | 0.038484239 | 0.140928198 | Map2k2 | http://amigo.geneontology.org/amigo/term/GO:0032872 |
| cytoskeletal anchoring at plasma membrane | Gene Ontology | GO:0007016 | 1 | 8 | 0.038484239 | 0.140928198 | Tln1 | http://amigo.geneontology.org/amigo/term/GO:0007016 |
| regulation of intracellular pH | Gene Ontology | GO:0051453 | 1 | 8 | 0.038484239 | 0.140928198 | Slc4a3 | http://amigo.geneontology.org/amigo/term/GO:0051453 |
| cardiac septum morphogenesis | Gene Ontology | GO:0060411 | 1 | 8 | 0.038484239 | 0.140928198 | Dhrs3 | http://amigo.geneontology.org/amigo/term/GO:0060411 |
| cellular carbohydrate metabolic process | Gene Ontology | GO:0044262 | 1 | 9 | 0.043191062 | 0.147758896 | Ldha | http://amigo.geneontology.org/amigo/term/GO:0044262 |
| cellular component organization | Gene Ontology | GO:0016043 | 1 | 9 | 0.043191062 | 0.147758896 | Fmnl3 | http://amigo.geneontology.org/amigo/term/GO:0016043 |
| synaptic vesicle transport | Gene Ontology | GO:0048489 | 1 | 9 | 0.043191062 | 0.147758896 | Snapin | http://amigo.geneontology.org/amigo/term/GO:0048489 |
| negative regulation of growth | Gene Ontology | GO:0045926 | 1 | 9 | 0.043191062 | 0.147758896 | Bbc3 | http://amigo.geneontology.org/amigo/term/GO:0045926 |
| mitochondrial fragmentation involved in apoptotic process | Gene Ontology | GO:0043653 | 1 | 9 | 0.043191062 | 0.147758896 | Dnm1l | http://amigo.geneontology.org/amigo/term/GO:0043653 |
| dephosphorylation | Gene Ontology | GO:0016311 | 3 | 164 | 0.046241114 | 0.149970515 | Ppap2c\|Nt5m\|Inpp4a | http://amigo.geneontology.org/amigo/term/GO:0016311 |
| negative regulation of translational initiation | Gene Ontology | GO:0045947 | 1 | 10 | 0.047875203 | 0.149970515 | Eif4ebp2 | http://amigo.geneontology.org/amigo/term/GO:0045947 |
| retinoid metabolic process | Gene Ontology | GO:0001523 | 1 | 10 | 0.047875203 | 0.149970515 | Dhrs3 | http://amigo.geneontology.org/amigo/term/GO:0001523 |
| G-protein coupled receptor internalization | Gene Ontology | GO:0002031 | 1 | 10 | 0.047875203 | 0.149970515 | Gtf2h2 | http://amigo.geneontology.org/amigo/term/GO:0002031 |
| embryonic heart tube morphogenesis | Gene Ontology | GO:0003143 | 1 | 10 | 0.047875203 | 0.149970515 | Tead1 | http://amigo.geneontology.org/amigo/term/GO:0003143 |
| cellular response to fibroblast growth factor stimulus | Gene Ontology | GO:0044344 | 1 | 10 | 0.047875203 | 0.149970515 | Cd44 | http://amigo.geneontology.org/amigo/term/GO:0044344 |
| paraxial mesoderm development | Gene Ontology | GO:0048339 | 1 | 10 | 0.047875203 | 0.149970515 | Tead1 | http://amigo.geneontology.org/amigo/term/GO:0048339 |
| mRNA transport | Gene Ontology | GO:0051028 | 2 | 74 | 0.050813973 | 0.151772887 | G3bp2\|Eif5a | http://amigo.geneontology.org/amigo/term/GO:0051028 |
| glutathione biosynthetic process | Gene Ontology | GO:0006750 | 1 | 11 | 0.052536769 | 0.151772887 | Haghl | http://amigo.geneontology.org/amigo/term/GO:0006750 |
| cardiac epithelial to mesenchymal transition | Gene Ontology | GO:0060317 | 1 | 11 | 0.052536769 | 0.151772887 | Adam15 | http://amigo.geneontology.org/amigo/term/GO:0060317 |
| phosphatidylinositol-3-phosphate biosynthetic process | Gene Ontology | GO:0036092 | 1 | 11 | 0.052536769 | 0.151772887 | Inpp4a | http://amigo.geneontology.org/amigo/term/GO:0036092 |
| ubiquinone biosynthetic process | Gene Ontology | GO:0006744 | 1 | 11 | 0.052536769 | 0.151772887 | Coq2 | http://amigo.geneontology.org/amigo/term/GO:0006744 |
| Leydig cell differentiation | Gene Ontology | GO:0033327 | 1 | 11 | 0.052536769 | 0.151772887 | Sf1 | http://amigo.geneontology.org/amigo/term/GO:0033327 |
| male sex determination | Gene Ontology | GO:0030238 | 1 | 11 | 0.052536769 | 0.151772887 | Sf1 | http://amigo.geneontology.org/amigo/term/GO:0030238 |
| negative regulation of DNA damage response, signal transduction by p53 class mediator | Gene Ontology | GO:0043518 | 1 | 12 | 0.057175866 | 0.156481318 | Cd44 | http://amigo.geneontology.org/amigo/term/GO:0043518 |
| regulation of cell size | Gene Ontology | GO:0008361 | 1 | 12 | 0.057175866 | 0.156481318 | Wdtc1 | http://amigo.geneontology.org/amigo/term/GO:0008361 |
| positive regulation of receptor-mediated endocytosis | Gene Ontology | GO:0048260 | 1 | 12 | 0.057175866 | 0.156481318 | Hnrnpk | http://amigo.geneontology.org/amigo/term/GO:0048260 |
| mitochondrial fission | Gene Ontology | GO:0000266 | 1 | 12 | 0.057175866 | 0.156481318 | Dnm1l | http://amigo.geneontology.org/amigo/term/GO:0000266 |
| I-kappaB phosphorylation | Gene Ontology | GO:0007252 | 1 | 12 | 0.057175866 | 0.156481318 | Ikbke | http://amigo.geneontology.org/amigo/term/GO:0007252 |
| positive regulation of Ras protein signal transduction | Gene Ontology | GO:0046579 | 1 | 13 | 0.061792603 | 0.162283603 | Shoc2 | http://amigo.geneontology.org/amigo/term/GO:0046579 |
| regulation of ossification | Gene Ontology | GO:0030278 | 1 | 13 | 0.061792603 | 0.162283603 | Dhrs3 | http://amigo.geneontology.org/amigo/term/GO:0030278 |
| necroptosis | Gene Ontology | GO:0070266 | 1 | 13 | 0.061792603 | 0.162283603 | Dnm1l | http://amigo.geneontology.org/amigo/term/GO:0070266 |
| synaptic vesicle exocytosis | Gene Ontology | GO:0016079 | 1 | 13 | 0.061792603 | 0.162283603 | Snapin | http://amigo.geneontology.org/amigo/term/GO:0016079 |
| determination of adult lifespan | Gene Ontology | GO:0008340 | 1 | 14 | 0.066387084 | 0.170897443 | Bbc3 | http://amigo.geneontology.org/amigo/term/GO:0008340 |
| ATP synthesis coupled proton transport | Gene Ontology | GO:0015986 | 1 | 14 | 0.066387084 | 0.170897443 | Atp5c1 | http://amigo.geneontology.org/amigo/term/GO:0015986 |
| histone H4-K5 acetylation | Gene Ontology | GO:0043981 | 1 | 15 | 0.070959415 | 0.174051396 | Jade1 | http://amigo.geneontology.org/amigo/term/GO:0043981 |
| histone H4-K8 acetylation | Gene Ontology | GO:0043982 | 1 | 15 | 0.070959415 | 0.174051396 | Jade1 | http://amigo.geneontology.org/amigo/term/GO:0043982 |
| isoprenoid biosynthetic process | Gene Ontology | GO:0008299 | 1 | 15 | 0.070959415 | 0.174051396 | Coq2 | http://amigo.geneontology.org/amigo/term/GO:0008299 |
| anterograde synaptic vesicle transport | Gene Ontology | GO:0048490 | 1 | 15 | 0.070959415 | 0.174051396 | Snapin | http://amigo.geneontology.org/amigo/term/GO:0048490 |
| cellular response to extracellular stimulus | Gene Ontology | GO:0031668 | 1 | 15 | 0.070959415 | 0.174051396 | Ldha | http://amigo.geneontology.org/amigo/term/GO:0031668 |
| execution phase of apoptosis | Gene Ontology | GO:0097194 | 1 | 16 | 0.075509702 | 0.181782616 | Bbc3 | http://amigo.geneontology.org/amigo/term/GO:0097194 |
| membrane fusion | Gene Ontology | GO:0061025 | 1 | 16 | 0.075509702 | 0.181782616 | Dnm1l | http://amigo.geneontology.org/amigo/term/GO:0061025 |
| hippo signaling cascade | Gene Ontology | GO:0035329 | 1 | 17 | 0.080038049 | 0.185802613 | Tead1 | http://amigo.geneontology.org/amigo/term/GO:0035329 |
| glycerol metabolic process | Gene Ontology | GO:0006071 | 1 | 17 | 0.080038049 | 0.185802613 | Coq2 | http://amigo.geneontology.org/amigo/term/GO:0006071 |
| response to ischemia | Gene Ontology | GO:0002931 | 1 | 17 | 0.080038049 | 0.185802613 | Hyou1 | http://amigo.geneontology.org/amigo/term/GO:0002931 |
| cortical actin cytoskeleton organization | Gene Ontology | GO:0030866 | 1 | 17 | 0.080038049 | 0.185802613 | Tln1 | http://amigo.geneontology.org/amigo/term/GO:0030866 |
| regulation of cell shape | Gene Ontology | GO:0008360 | 2 | 98 | 0.083063815 | 0.189496427 | Baiap2\|Fmnl3 | http://amigo.geneontology.org/amigo/term/GO:0008360 |
| mitochondrion morphogenesis | Gene Ontology | GO:0070584 | 1 | 18 | 0.08454456 | 0.189496427 | Dnm1l | http://amigo.geneontology.org/amigo/term/GO:0070584 |
| regulation of protein binding | Gene Ontology | GO:0043393 | 1 | 18 | 0.08454456 | 0.189496427 | Snapin | http://amigo.geneontology.org/amigo/term/GO:0043393 |
| negative regulation of G1/S transition of mitotic cell cycle | Gene Ontology | GO:2000134 | 1 | 18 | 0.08454456 | 0.189496427 | Jade1 | http://amigo.geneontology.org/amigo/term/GO:2000134 |
| filopodium assembly | Gene Ontology | GO:0046847 | 1 | 19 | 0.089029338 | 0.194464375 | Baiap2 | http://amigo.geneontology.org/amigo/term/GO:0046847 |
| behavioral response to pain | Gene Ontology | GO:0048266 | 1 | 19 | 0.089029338 | 0.194464375 | Git2 | http://amigo.geneontology.org/amigo/term/GO:0048266 |
| neuron projection development | Gene Ontology | GO:0031175 | 2 | 105 | 0.093401603 | 0.194464375 | Cd44\|Snapin | http://amigo.geneontology.org/amigo/term/GO:0031175 |
| peroxisome organization | Gene Ontology | GO:0007031 | 1 | 20 | 0.093492488 | 0.194464375 | Acot8 | http://amigo.geneontology.org/amigo/term/GO:0007031 |
| anterograde axon cargo transport | Gene Ontology | GO:0008089 | 1 | 20 | 0.093492488 | 0.194464375 | Snapin | http://amigo.geneontology.org/amigo/term/GO:0008089 |
| cAMP-mediated signaling | Gene Ontology | GO:0019933 | 1 | 20 | 0.093492488 | 0.194464375 | Eif4ebp2 | http://amigo.geneontology.org/amigo/term/GO:0019933 |
| regulation of translational initiation | Gene Ontology | GO:0006446 | 1 | 20 | 0.093492488 | 0.194464375 | Eif4ebp2 | http://amigo.geneontology.org/amigo/term/GO:0006446 |
| nitrogen compound metabolic process | Gene Ontology | GO:0006807 | 1 | 20 | 0.093492488 | 0.194464375 | Nit1 | http://amigo.geneontology.org/amigo/term/GO:0006807 |
| androgen receptor signaling pathway | Gene Ontology | GO:0030521 | 1 | 20 | 0.093492488 | 0.194464375 | Dnaja1 | http://amigo.geneontology.org/amigo/term/GO:0030521 |
| RNA splicing | Gene Ontology | GO:0008380 | 3 | 222 | 0.094772737 | 0.19556279 | Hnrnpk\|Hnrnpf\|Sf1 | http://amigo.geneontology.org/amigo/term/GO:0008380 |
| collagen catabolic process | Gene Ontology | GO:0030574 | 1 | 22 | 0.10235431 | 0.201606974 | Adam15 | http://amigo.geneontology.org/amigo/term/GO:0030574 |
| mRNA export from nucleus | Gene Ontology | GO:0006406 | 1 | 22 | 0.10235431 | 0.201606974 | Eif5a | http://amigo.geneontology.org/amigo/term/GO:0006406 |
| protein export from nucleus | Gene Ontology | GO:0006611 | 1 | 22 | 0.10235431 | 0.201606974 | Eif5a | http://amigo.geneontology.org/amigo/term/GO:0006611 |
| regulation of ARF GTPase activity | Gene Ontology | GO:0032312 | 1 | 22 | 0.10235431 | 0.201606974 | Git2 | http://amigo.geneontology.org/amigo/term/GO:0032312 |
| programmed cell death | Gene Ontology | GO:0012501 | 1 | 22 | 0.10235431 | 0.201606974 | Dnm1l | http://amigo.geneontology.org/amigo/term/GO:0012501 |
| positive regulation of protein serine/threonine kinase activity | Gene Ontology | GO:0071902 | 1 | 22 | 0.10235431 | 0.201606974 | Map2k2 | http://amigo.geneontology.org/amigo/term/GO:0071902 |
| response to stress | Gene Ontology | GO:0006950 | 2 | 113 | 0.105645404 | 0.205598729 | Ahsa2\|Hyou1 | http://amigo.geneontology.org/amigo/term/GO:0006950 |
| response to DNA damage stimulus | Gene Ontology | GO:0006974 | 4 | 369 | 0.106286644 | 0.205598729 | Gtf2h2\|Bbc3\|2310003H01Rik\|Ikbke | http://amigo.geneontology.org/amigo/term/GO:0006974 |
| cellular response to ionizing radiation | Gene Ontology | GO:0071479 | 1 | 23 | 0.106753186 | 0.205598729 | Bbc3 | http://amigo.geneontology.org/amigo/term/GO:0071479 |
| regulation of RNA splicing | Gene Ontology | GO:0043484 | 1 | 24 | 0.111130841 | 0.205662449 | Hnrnpf | http://amigo.geneontology.org/amigo/term/GO:0043484 |
| I-kappaB kinase/NF-kappaB cascade | Gene Ontology | GO:0007249 | 1 | 24 | 0.111130841 | 0.205662449 | Snip1 | http://amigo.geneontology.org/amigo/term/GO:0007249 |
| cytokine production | Gene Ontology | GO:0001816 | 1 | 24 | 0.111130841 | 0.205662449 | Nfat5 | http://amigo.geneontology.org/amigo/term/GO:0001816 |
| mitotic cell cycle checkpoint | Gene Ontology | GO:0007093 | 1 | 24 | 0.111130841 | 0.205662449 | Pura | http://amigo.geneontology.org/amigo/term/GO:0007093 |
| actin filament bundle assembly | Gene Ontology | GO:0051017 | 1 | 24 | 0.111130841 | 0.205662449 | Baiap2 | http://amigo.geneontology.org/amigo/term/GO:0051017 |
| acyl-CoA metabolic process | Gene Ontology | GO:0006637 | 1 | 25 | 0.115487375 | 0.205662449 | Acot8 | http://amigo.geneontology.org/amigo/term/GO:0006637 |
| ATP metabolic process | Gene Ontology | GO:0046034 | 1 | 25 | 0.115487375 | 0.205662449 | Atp5c1 | http://amigo.geneontology.org/amigo/term/GO:0046034 |
| signal transduction by p53 class mediator resulting in induction of apoptosis | Gene Ontology | GO:0072332 | 1 | 25 | 0.115487375 | 0.205662449 | Bbc3 | http://amigo.geneontology.org/amigo/term/GO:0072332 |
| ATP biosynthetic process | Gene Ontology | GO:0006754 | 1 | 25 | 0.115487375 | 0.205662449 | Atp5c1 | http://amigo.geneontology.org/amigo/term/GO:0006754 |
| intrinsic apoptotic signaling pathway in response to endoplasmic reticulum stress | Gene Ontology | GO:0070059 | 1 | 25 | 0.115487375 | 0.205662449 | Bbc3 | http://amigo.geneontology.org/amigo/term/GO:0070059 |
| apoptotic mitochondrial changes | Gene Ontology | GO:0008637 | 1 | 25 | 0.115487375 | 0.205662449 | Dap3 | http://amigo.geneontology.org/amigo/term/GO:0008637 |
| apoptotic process | Gene Ontology | GO:0006915 | 5 | 531 | 0.117611871 | 0.207693008 | Bbc3\|Dap3\|Pura\|Jade1\|Eif5a | http://amigo.geneontology.org/amigo/term/GO:0006915 |
| response to bacterium | Gene Ontology | GO:0009617 | 1 | 26 | 0.119822889 | 0.207693008 | Baiap2 | http://amigo.geneontology.org/amigo/term/GO:0009617 |
| synapse assembly | Gene Ontology | GO:0007416 | 1 | 26 | 0.119822889 | 0.207693008 | Farp1 | http://amigo.geneontology.org/amigo/term/GO:0007416 |
| anion transport | Gene Ontology | GO:0006820 | 1 | 26 | 0.119822889 | 0.207693008 | Slc4a3 | http://amigo.geneontology.org/amigo/term/GO:0006820 |
| positive regulation of protein secretion | Gene Ontology | GO:0050714 | 1 | 27 | 0.124137483 | 0.212340432 | Dnm1l | http://amigo.geneontology.org/amigo/term/GO:0050714 |
| nucleotide metabolic process | Gene Ontology | GO:0009117 | 1 | 27 | 0.124137483 | 0.212340432 | Nt5m | http://amigo.geneontology.org/amigo/term/GO:0009117 |
| translational elongation | Gene Ontology | GO:0006414 | 1 | 28 | 0.128431256 | 0.215433074 | Eif5a | http://amigo.geneontology.org/amigo/term/GO:0006414 |
| release of sequestered calcium ion into cytosol | Gene Ontology | GO:0051209 | 1 | 28 | 0.128431256 | 0.215433074 | Bbc3 | http://amigo.geneontology.org/amigo/term/GO:0051209 |
| bone morphogenesis | Gene Ontology | GO:0060349 | 1 | 28 | 0.128431256 | 0.215433074 | Dhrs3 | http://amigo.geneontology.org/amigo/term/GO:0060349 |
| regulation of Rac GTPase activity | Gene Ontology | GO:0032314 | 1 | 29 | 0.132704306 | 0.219765093 | Farp1 | http://amigo.geneontology.org/amigo/term/GO:0032314 |
| DNA damage response, signal transduction by p53 class mediator resulting in induction of apoptosis | Gene Ontology | GO:0042771 | 1 | 29 | 0.132704306 | 0.219765093 | Bbc3 | http://amigo.geneontology.org/amigo/term/GO:0042771 |
| nucleotide-excision repair | Gene Ontology | GO:0006289 | 1 | 30 | 0.136956733 | 0.223835638 | Gtf2h2 | http://amigo.geneontology.org/amigo/term/GO:0006289 |
| positive regulation of apoptotic process | Gene Ontology | GO:0043065 | 3 | 265 | 0.139984856 | 0.223835638 | Dnm1l\|Bbc3\|Eif5a | http://amigo.geneontology.org/amigo/term/GO:0043065 |
| response to heat | Gene Ontology | GO:0009408 | 1 | 31 | 0.141188634 | 0.223835638 | Dnaja1 | http://amigo.geneontology.org/amigo/term/GO:0009408 |
| glycolysis | Gene Ontology | GO:0006096 | 1 | 31 | 0.141188634 | 0.223835638 | Ldha | http://amigo.geneontology.org/amigo/term/GO:0006096 |
| dendrite development | Gene Ontology | GO:0016358 | 1 | 31 | 0.141188634 | 0.223835638 | Baiap2 | http://amigo.geneontology.org/amigo/term/GO:0016358 |
| dendrite morphogenesis | Gene Ontology | GO:0048813 | 1 | 31 | 0.141188634 | 0.223835638 | Farp1 | http://amigo.geneontology.org/amigo/term/GO:0048813 |
| regulation of actin cytoskeleton organization | Gene Ontology | GO:0032956 | 1 | 31 | 0.141188634 | 0.223835638 | Baiap2 | http://amigo.geneontology.org/amigo/term/GO:0032956 |
| cell migration | Gene Ontology | GO:0016477 | 2 | 138 | 0.146323355 | 0.230570135 | Cd44\|Fmnl3 | http://amigo.geneontology.org/amigo/term/GO:0016477 |
| apoptotic signaling pathway | Gene Ontology | GO:0097190 | 1 | 33 | 0.149591247 | 0.234299544 | Bbc3 | http://amigo.geneontology.org/amigo/term/GO:0097190 |
| regulation of synaptic plasticity | Gene Ontology | GO:0048167 | 1 | 34 | 0.153762154 | 0.237965238 | Baiap2 | http://amigo.geneontology.org/amigo/term/GO:0048167 |
| signal transduction by phosphorylation | Gene Ontology | GO:0023014 | 1 | 34 | 0.153762154 | 0.237965238 | Ikbke | http://amigo.geneontology.org/amigo/term/GO:0023014 |
| negative regulation of smooth muscle cell proliferation | Gene Ontology | GO:0048662 | 1 | 35 | 0.157912922 | 0.242942957 | Sf1 | http://amigo.geneontology.org/amigo/term/GO:0048662 |
| Rho protein signal transduction | Gene Ontology | GO:0007266 | 1 | 36 | 0.162043647 | 0.24783146 | Baiap2 | http://amigo.geneontology.org/amigo/term/GO:0007266 |
| histone H3 acetylation | Gene Ontology | GO:0043966 | 1 | 37 | 0.166154425 | 0.252632459 | Jade1 | http://amigo.geneontology.org/amigo/term/GO:0043966 |
| mRNA processing | Gene Ontology | GO:0006397 | 3 | 295 | 0.175100525 | 0.26468684 | Hnrnpk\|Hnrnpf\|Sf1 | http://amigo.geneontology.org/amigo/term/GO:0006397 |
| regulation of cell growth | Gene Ontology | GO:0001558 | 1 | 40 | 0.178368019 | 0.268067543 | Cd44 | http://amigo.geneontology.org/amigo/term/GO:0001558 |
| outflow tract morphogenesis | Gene Ontology | GO:0003151 | 1 | 44 | 0.194379246 | 0.290451747 | Dhrs3 | http://amigo.geneontology.org/amigo/term/GO:0003151 |
| branching involved in ureteric bud morphogenesis | Gene Ontology | GO:0001658 | 1 | 45 | 0.198333819 | 0.292993142 | Cd44 | http://amigo.geneontology.org/amigo/term/GO:0001658 |
| insulin receptor signaling pathway | Gene Ontology | GO:0008286 | 1 | 45 | 0.198333819 | 0.292993142 | Eif4ebp2 | http://amigo.geneontology.org/amigo/term/GO:0008286 |
| biosynthetic process | Gene Ontology | GO:0009058 | 1 | 46 | 0.202269283 | 0.295449514 | Coq2 | http://amigo.geneontology.org/amigo/term/GO:0009058 |
| response to endoplasmic reticulum stress | Gene Ontology | GO:0034976 | 1 | 46 | 0.202269283 | 0.295449514 | Bbc3 | http://amigo.geneontology.org/amigo/term/GO:0034976 |
| sperm motility | Gene Ontology | GO:0030317 | 1 | 47 | 0.206185727 | 0.299487648 | Dnaja1 | http://amigo.geneontology.org/amigo/term/GO:0030317 |
| gene silencing by RNA | Gene Ontology | GO:0031047 | 1 | 48 | 0.210083242 | 0.301777033 | Snip1 | http://amigo.geneontology.org/amigo/term/GO:0031047 |
| negative regulation of phosphatase activity | Gene Ontology | GO:0010923 | 1 | 48 | 0.210083242 | 0.301777033 | Farp1 | http://amigo.geneontology.org/amigo/term/GO:0010923 |
| ATP catabolic process | Gene Ontology | GO:0006200 | 2 | 179 | 0.218196487 | 0.311709267 | Atp5c1\|Gtf2h2 | http://amigo.geneontology.org/amigo/term/GO:0006200 |
| protein ubiquitination | Gene Ontology | GO:0016567 | 2 | 180 | 0.219996376 | 0.312563157 | Wdsub1\|Rlim | http://amigo.geneontology.org/amigo/term/GO:0016567 |
| DNA damage response, signal transduction resulting in induction of apoptosis | Gene Ontology | GO:0008630 | 1 | 52 | 0.225485811 | 0.318621255 | Ikbke | http://amigo.geneontology.org/amigo/term/GO:0008630 |
| positive regulation of neuron apoptotic process | Gene Ontology | GO:0043525 | 1 | 53 | 0.229290025 | 0.322245441 | Bbc3 | http://amigo.geneontology.org/amigo/term/GO:0043525 |
| phospholipid biosynthetic process | Gene Ontology | GO:0008654 | 1 | 54 | 0.233075844 | 0.324062671 | Agpat3 | http://amigo.geneontology.org/amigo/term/GO:0008654 |
| stem cell maintenance | Gene Ontology | GO:0019827 | 1 | 54 | 0.233075844 | 0.324062671 | Med7 | http://amigo.geneontology.org/amigo/term/GO:0019827 |
| intracellular protein transport | Gene Ontology | GO:0006886 | 2 | 188 | 0.234439779 | 0.324225227 | Tnpo1\|Snapin | http://amigo.geneontology.org/amigo/term/GO:0006886 |
| negative regulation of catalytic activity | Gene Ontology | GO:0043086 | 1 | 56 | 0.240592648 | 0.327508317 | Wdtc1 | http://amigo.geneontology.org/amigo/term/GO:0043086 |
| proton transport | Gene Ontology | GO:0015992 | 1 | 56 | 0.240592648 | 0.327508317 | Atp5c1 | http://amigo.geneontology.org/amigo/term/GO:0015992 |
| cellular response to insulin stimulus | Gene Ontology | GO:0032869 | 1 | 56 | 0.240592648 | 0.327508317 | Wdtc1 | http://amigo.geneontology.org/amigo/term/GO:0032869 |
| positive regulation of peptidyl-serine phosphorylation | Gene Ontology | GO:0033138 | 1 | 57 | 0.244323807 | 0.32914088 | Cd44 | http://amigo.geneontology.org/amigo/term/GO:0033138 |
| protein homotetramerization | Gene Ontology | GO:0051289 | 1 | 57 | 0.244323807 | 0.32914088 | Dnm1l | http://amigo.geneontology.org/amigo/term/GO:0051289 |
| negative regulation of sequence-specific DNA binding transcription factor activity | Gene Ontology | GO:0043433 | 1 | 58 | 0.248036918 | 0.332420612 | Rlim | http://amigo.geneontology.org/amigo/term/GO:0043433 |
| positive regulation of gene expression | Gene Ontology | GO:0010628 | 2 | 197 | 0.250758569 | 0.333930295 | Nfat5\|Cd44 | http://amigo.geneontology.org/amigo/term/GO:0010628 |
| steroid biosynthetic process | Gene Ontology | GO:0006694 | 1 | 59 | 0.251732068 | 0.333930295 | Tecr | http://amigo.geneontology.org/amigo/term/GO:0006694 |
| glucose metabolic process | Gene Ontology | GO:0006006 | 1 | 60 | 0.255409343 | 0.337088473 | Wdtc1 | http://amigo.geneontology.org/amigo/term/GO:0006006 |
| regulation of Rho protein signal transduction | Gene Ontology | GO:0035023 | 1 | 64 | 0.269941381 | 0.352687232 | Farp1 | http://amigo.geneontology.org/amigo/term/GO:0035023 |
| activation of MAPK activity | Gene Ontology | GO:0000187 | 1 | 64 | 0.269941381 | 0.352687232 | Map2k2 | http://amigo.geneontology.org/amigo/term/GO:0000187 |
| negative regulation of cysteine-type endopeptidase activity involved in apoptotic process | Gene Ontology | GO:0043154 | 1 | 67 | 0.280656847 | 0.364853901 | Cd44 | http://amigo.geneontology.org/amigo/term/GO:0043154 |
| protein transport | Gene Ontology | GO:0015031 | 4 | 557 | 0.289867545 | 0.372987876 | Trim3\|Tnpo1\|Timm10b\|Eif5a | http://amigo.geneontology.org/amigo/term/GO:0015031 |
| chromosome segregation | Gene Ontology | GO:0007059 | 1 | 70 | 0.291217457 | 0.372987876 | Pmf1 | http://amigo.geneontology.org/amigo/term/GO:0007059 |
| activation of cysteine-type endopeptidase activity involved in apoptotic process | Gene Ontology | GO:0006919 | 1 | 70 | 0.291217457 | 0.372987876 | Bbc3 | http://amigo.geneontology.org/amigo/term/GO:0006919 |
| negative regulation of apoptotic process | Gene Ontology | GO:0043066 | 3 | 393 | 0.301979679 | 0.384876062 | Naa16\|Cd44\|Eif5a | http://amigo.geneontology.org/amigo/term/GO:0043066 |
| protein stabilization | Gene Ontology | GO:0050821 | 1 | 75 | 0.308480322 | 0.387748831 | Naa16 | http://amigo.geneontology.org/amigo/term/GO:0050821 |
| palate development | Gene Ontology | GO:0060021 | 1 | 75 | 0.308480322 | 0.387748831 | Dhrs3 | http://amigo.geneontology.org/amigo/term/GO:0060021 |
| transcription from RNA polymerase II promoter | Gene Ontology | GO:0006366 | 3 | 398 | 0.308707723 | 0.387748831 | Gtf2h2\|Hnrnpk\|Jade1 | http://amigo.geneontology.org/amigo/term/GO:0006366 |
| MAPK cascade | Gene Ontology | GO:0000165 | 1 | 76 | 0.311882895 | 0.389853618 | Map2k2 | http://amigo.geneontology.org/amigo/term/GO:0000165 |
| protein heterooligomerization | Gene Ontology | GO:0051291 | 1 | 77 | 0.315268985 | 0.392200652 | Sept9 | http://amigo.geneontology.org/amigo/term/GO:0051291 |
| positive regulation of peptidyl-tyrosine phosphorylation | Gene Ontology | GO:0050731 | 1 | 78 | 0.318638673 | 0.394505023 | Cd44 | http://amigo.geneontology.org/amigo/term/GO:0050731 |
| regulation of RNA biosynthetic process | Gene Ontology | GO:2001141 | 1 | 79 | 0.321992035 | 0.396767437 | Pmf1 | http://amigo.geneontology.org/amigo/term/GO:2001141 |
| multicellular organism growth | Gene Ontology | GO:0035264 | 1 | 81 | 0.328650096 | 0.403061439 | Wdtc1 | http://amigo.geneontology.org/amigo/term/GO:0035264 |
| exocytosis | Gene Ontology | GO:0006887 | 1 | 86 | 0.345014965 | 0.41917706 | Snapin | http://amigo.geneontology.org/amigo/term/GO:0006887 |
| cytoskeleton organization | Gene Ontology | GO:0007010 | 1 | 86 | 0.345014965 | 0.41917706 | Fmnl3 | http://amigo.geneontology.org/amigo/term/GO:0007010 |
| cell adhesion | Gene Ontology | GO:0007155 | 3 | 452 | 0.381406438 | 0.460664083 | Cd44\|Adam15\|Tln1 | http://amigo.geneontology.org/amigo/term/GO:0007155 |
| response to hypoxia | Gene Ontology | GO:0001666 | 1 | 98 | 0.382705546 | 0.460664083 | Hyou1 | http://amigo.geneontology.org/amigo/term/GO:0001666 |
| positive regulation of ERK1 and ERK2 cascade | Gene Ontology | GO:0070374 | 1 | 104 | 0.400741876 | 0.480151557 | Cd44 | http://amigo.geneontology.org/amigo/term/GO:0070374 |
| oxidation-reduction process | Gene Ontology | GO:0055114 | 4 | 662 | 0.407524929 | 0.486038906 | Kdm4b\|Dhrs3\|Tecr\|Ldha | http://amigo.geneontology.org/amigo/term/GO:0055114 |
| regulation of translation | Gene Ontology | GO:0006417 | 1 | 109 | 0.415375068 | 0.492069661 | Eif4ebp2 | http://amigo.geneontology.org/amigo/term/GO:0006417 |
| DNA repair | Gene Ontology | GO:0006281 | 2 | 290 | 0.416567346 | 0.492069661 | Gtf2h2\|2310003H01Rik | http://amigo.geneontology.org/amigo/term/GO:0006281 |
| negative regulation of cell growth | Gene Ontology | GO:0030308 | 1 | 110 | 0.418259212 | 0.492069661 | Bbc3 | http://amigo.geneontology.org/amigo/term/GO:0030308 |
| protein folding | Gene Ontology | GO:0006457 | 1 | 112 | 0.423985544 | 0.496559647 | Dnaja1 | http://amigo.geneontology.org/amigo/term/GO:0006457 |
| synaptic transmission | Gene Ontology | GO:0007268 | 1 | 124 | 0.457196508 | 0.533054224 | Snapin | http://amigo.geneontology.org/amigo/term/GO:0007268 |
| regulation of transcription from RNA polymerase II promoter | Gene Ontology | GO:0006357 | 2 | 317 | 0.461693196 | 0.535893889 | Med7\|Pknox2 | http://amigo.geneontology.org/amigo/term/GO:0006357 |
| actin cytoskeleton organization | Gene Ontology | GO:0030036 | 1 | 128 | 0.467842244 | 0.540617705 | Fmnl3 | http://amigo.geneontology.org/amigo/term/GO:0030036 |
| ubiquitin-dependent protein catabolic process | Gene Ontology | GO:0006511 | 1 | 131 | 0.475691413 | 0.547255608 | Rlim | http://amigo.geneontology.org/amigo/term/GO:0006511 |
| ion transmembrane transport | Gene Ontology | GO:0034220 | 1 | 139 | 0.49606966 | 0.568185513 | Slc4a3 | http://amigo.geneontology.org/amigo/term/GO:0034220 |
| cell division | Gene Ontology | GO:0051301 | 2 | 344 | 0.50478947 | 0.575637115 | Sept9\|Pmf1 | http://amigo.geneontology.org/amigo/term/GO:0051301 |
| regulation of cell proliferation | Gene Ontology | GO:0042127 | 1 | 144 | 0.508408009 | 0.5772318 | Tead1 | http://amigo.geneontology.org/amigo/term/GO:0042127 |
| positive regulation of transcription from RNA polymerase II promoter | Gene Ontology | GO:0045944 | 4 | 758 | 0.512329407 | 0.579154982 | Nfat5\|Tead1\|Hnrnpk\|Jade1 | http://amigo.geneontology.org/amigo/term/GO:0045944 |
| endocytosis | Gene Ontology | GO:0006897 | 1 | 149 | 0.520448869 | 0.585786605 | Dnm1l | http://amigo.geneontology.org/amigo/term/GO:0006897 |
| GTP catabolic process | Gene Ontology | GO:0006184 | 1 | 158 | 0.54139518 | 0.606735978 | Dnm1l | http://amigo.geneontology.org/amigo/term/GO:0006184 |
| protein phosphorylation | Gene Ontology | GO:0006468 | 3 | 584 | 0.549106307 | 0.612736652 | Gtf2h2\|Map2k2\|Ikbke | http://amigo.geneontology.org/amigo/term/GO:0006468 |
| cell cycle | Gene Ontology | GO:0007049 | 3 | 590 | 0.556134702 | 0.616323581 | Sept9\|Pmf1\|Foxn3 | http://amigo.geneontology.org/amigo/term/GO:0007049 |
| protein homooligomerization | Gene Ontology | GO:0051260 | 1 | 165 | 0.557061698 | 0.616323581 | Lzts3 | http://amigo.geneontology.org/amigo/term/GO:0051260 |
| heart development | Gene Ontology | GO:0007507 | 1 | 167 | 0.561440256 | 0.618535875 | Tead1 | http://amigo.geneontology.org/amigo/term/GO:0007507 |
| positive regulation of cell proliferation | Gene Ontology | GO:0008284 | 2 | 393 | 0.57723243 | 0.633250767 | Pura\|Eif5a | http://amigo.geneontology.org/amigo/term/GO:0008284 |
| cell proliferation | Gene Ontology | GO:0008283 | 1 | 180 | 0.588880228 | 0.643314535 | Pura | http://amigo.geneontology.org/amigo/term/GO:0008283 |
| carbohydrate metabolic process | Gene Ontology | GO:0005975 | 1 | 187 | 0.602948194 | 0.653193877 | Ldha | http://amigo.geneontology.org/amigo/term/GO:0005975 |
| Wnt receptor signaling pathway | Gene Ontology | GO:0016055 | 1 | 187 | 0.602948194 | 0.653193877 | Cd44 | http://amigo.geneontology.org/amigo/term/GO:0016055 |
| transport | Gene Ontology | GO:0006810 | 8 | 1725 | 0.620382274 | 0.667764698 | Atp5c1\|Slc25a51\|Tnpo1\|Timm10b\|Slc4a3\|G3bp2\|Trim3\|Eif5a | http://amigo.geneontology.org/amigo/term/GO:0006810 |
| lipid metabolic process | Gene Ontology | GO:0006629 | 2 | 426 | 0.621606406 | 0.667764698 | Agpat3\|Tecr | http://amigo.geneontology.org/amigo/term/GO:0006629 |
| angiogenesis | Gene Ontology | GO:0001525 | 1 | 198 | 0.62410316 | 0.667764698 | Adam15 | http://amigo.geneontology.org/amigo/term/GO:0001525 |
| phosphorylation | Gene Ontology | GO:0016310 | 3 | 665 | 0.638422785 | 0.680286574 | Map2k2\|Git2\|Ikbke | http://amigo.geneontology.org/amigo/term/GO:0016310 |
| small GTPase mediated signal transduction | Gene Ontology | GO:0007264 | 1 | 217 | 0.658060917 | 0.698350361 | G3bp2 | http://amigo.geneontology.org/amigo/term/GO:0007264 |
| translation | Gene Ontology | GO:0006412 | 1 | 240 | 0.695147013 | 0.734708225 | Eif5a | http://amigo.geneontology.org/amigo/term/GO:0006412 |
| in utero embryonic development | Gene Ontology | GO:0001701 | 1 | 245 | 0.702669233 | 0.739651824 | Wdtc1 | http://amigo.geneontology.org/amigo/term/GO:0001701 |
| mitosis | Gene Ontology | GO:0007067 | 1 | 256 | 0.718580878 | 0.753350921 | Pmf1 | http://amigo.geneontology.org/amigo/term/GO:0007067 |
| chromatin modification | Gene Ontology | GO:0016568 | 1 | 259 | 0.722772947 | 0.754702676 | Kdm4b | http://amigo.geneontology.org/amigo/term/GO:0016568 |
| negative regulation of transcription from RNA polymerase II promoter | Gene Ontology | GO:0000122 | 2 | 556 | 0.762364186 | 0.787976899 | Rlim\|Wdtc1 | http://amigo.geneontology.org/amigo/term/GO:0000122 |
| metabolic process | Gene Ontology | GO:0008152 | 2 | 556 | 0.762364186 | 0.787976899 | Agpat3\|Dhrs3 | http://amigo.geneontology.org/amigo/term/GO:0008152 |
| spermatogenesis | Gene Ontology | GO:0007283 | 1 | 292 | 0.765016917 | 0.787976899 | Dnaja1 | http://amigo.geneontology.org/amigo/term/GO:0007283 |
| ion transport | Gene Ontology | GO:0006811 | 2 | 561 | 0.766762136 | 0.787976899 | Atp5c1\|Slc4a3 | http://amigo.geneontology.org/amigo/term/GO:0006811 |
| nervous system development | Gene Ontology | GO:0007399 | 1 | 325 | 0.800908997 | 0.819828107 | Pura | http://amigo.geneontology.org/amigo/term/GO:0007399 |
| intracellular signal transduction | Gene Ontology | GO:0035556 | 1 | 356 | 0.829682549 | 0.845950834 | Spsb2 | http://amigo.geneontology.org/amigo/term/GO:0035556 |
| negative regulation of transcription, DNA-dependent | Gene Ontology | GO:0045892 | 1 | 408 | 0.869030522 | 0.882609124 | Pura | http://amigo.geneontology.org/amigo/term/GO:0045892 |
| proteolysis | Gene Ontology | GO:0006508 | 1 | 549 | 0.936109564 | 0.947036913 | Adam15 | http://amigo.geneontology.org/amigo/term/GO:0006508 |
| cell differentiation | Gene Ontology | GO:0030154 | 1 | 671 | 0.965891871 | 0.973379404 | Pura | http://amigo.geneontology.org/amigo/term/GO:0030154 |
| multicellular organismal development | Gene Ontology | GO:0007275 | 1 | 987 | 0.993481456 | 0.996895239 | Farp1 | http://amigo.geneontology.org/amigo/term/GO:0007275 |
| signal transduction | Gene Ontology | GO:0007165 | 1 | 1126 | 0.996895239 | 0.996895239 | Baiap2 | http://amigo.geneontology.org/amigo/term/GO:0007165 |

KEGG pathway

| **#Term** | **Database** | **ID** | **Input number** | **Background number** | **P-Value** | **Corrected P-Value** | **Input** | **Hyperlink** |
| --- | --- | --- | --- | --- | --- | --- | --- | --- |
| Metabolic pathways | KEGG PATHWAY | mmu01100 | 10 | 1226 | 0.008192968 | 0.377879789 | Atp5c1\|Ppap2c\|Dhrs3\|Inpp4a\|Tcirg1\|Agpat3\|Acot8\|Nt5m\|Ldha\|Coq2 | http://www.genome.jp/kegg-bin/show_pathway?mmu01100/mmu:28169%09red/mmu:27060%09red/mmu:16828%09red/mmu:103850%09red/mmu:50784%09red/mmu:170789%09red/mmu:11949%09red/mmu:269180%09red/mmu:71883%09red/mmu:20148%09red |
| Pyruvate metabolism | KEGG PATHWAY | mmu00620 | 2 | 42 | 0.010017565 | 0.377879789 | Haghl\|Ldha | http://www.genome.jp/kegg-bin/show_pathway?mmu00620/mmu:68977%09red/mmu:16828%09red |
| Glycerolipid metabolism | KEGG PATHWAY | mmu00561 | 2 | 56 | 0.01736747 | 0.377879789 | Ppap2c\|Agpat3 | http://www.genome.jp/kegg-bin/show_pathway?mmu00561/mmu:28169%09red/mmu:50784%09red |
| Ubiquinone and other terpenoid-quinone biosynthesis | KEGG PATHWAY | mmu00130 | 1 | 10 | 0.035781445 | 0.377879789 | Coq2 | http://www.genome.jp/kegg-bin/show_pathway?mmu00130/mmu:71883%09red |
| Glycerophospholipid metabolism | KEGG PATHWAY | mmu00564 | 2 | 91 | 0.042734136 | 0.377879789 | Ppap2c\|Agpat3 | http://www.genome.jp/kegg-bin/show_pathway?mmu00564/mmu:28169%09red/mmu:50784%09red |
| Toll-like receptor signaling pathway | KEGG PATHWAY | mmu04620 | 2 | 101 | 0.051550058 | 0.377879789 | Map2k2\|Ikbke | http://www.genome.jp/kegg-bin/show_pathway?mmu04620/mmu:56489%09red/mmu:26396%09red |
| Primary bile acid biosynthesis | KEGG PATHWAY | mmu00120 | 1 | 16 | 0.056655081 | 0.377879789 | Acot8 | http://www.genome.jp/kegg-bin/show_pathway?mmu00120/mmu:170789%09red |
| HIF-1 signaling pathway | KEGG PATHWAY | mmu04066 | 2 | 112 | 0.06193416 | 0.377879789 | Map2k2\|Ldha | http://www.genome.jp/kegg-bin/show_pathway?mmu04066/mmu:16828%09red/mmu:26396%09red |
| MicroRNAs in cancer | KEGG PATHWAY | mmu05206 | 3 | 269 | 0.072481796 | 0.377879789 | Map2k2\|Hnrnpk\|Cd44 | http://www.genome.jp/kegg-bin/show_pathway?mmu05206/mmu:15387%09red/mmu:26396%09red/mmu:12505%09red |
| Fatty acid elongation | KEGG PATHWAY | mmu00062 | 1 | 24 | 0.083810681 | 0.377879789 | Tecr | http://www.genome.jp/kegg-bin/show_pathway?mmu00062/mmu:106529%09red |
| Oxidative phosphorylation | KEGG PATHWAY | mmu00190 | 2 | 136 | 0.086775593 | 0.377879789 | Atp5c1\|Tcirg1 | http://www.genome.jp/kegg-bin/show_pathway?mmu00190/mmu:27060%09red/mmu:11949%09red |
| Biosynthesis of unsaturated fatty acids | KEGG PATHWAY | mmu01040 | 1 | 25 | 0.087151728 | 0.377879789 | Tecr | http://www.genome.jp/kegg-bin/show_pathway?mmu01040/mmu:106529%09red |
| Measles | KEGG PATHWAY | mmu05162 | 2 | 139 | 0.090067705 | 0.377879789 | Bbc3\|Ikbke | http://www.genome.jp/kegg-bin/show_pathway?mmu05162/mmu:56489%09red/mmu:170770%09red |
| Collecting duct acid secretion | KEGG PATHWAY | mmu04966 | 1 | 27 | 0.093798659 | 0.377879789 | Tcirg1 | http://www.genome.jp/kegg-bin/show_pathway?mmu04966/mmu:27060%09red |
| Hepatitis B | KEGG PATHWAY | mmu05161 | 2 | 147 | 0.099027397 | 0.377879789 | Map2k2\|Ikbke | http://www.genome.jp/kegg-bin/show_pathway?mmu05161/mmu:56489%09red/mmu:26396%09red |
| Thyroid cancer | KEGG PATHWAY | mmu05216 | 1 | 29 | 0.100398971 | 0.377879789 | Map2k2 | http://www.genome.jp/kegg-bin/show_pathway?mmu05216/mmu:26396%09red |
| Nicotinate and nicotinamide metabolism | KEGG PATHWAY | mmu00760 | 1 | 31 | 0.106952977 | 0.377879789 | Nt5m | http://www.genome.jp/kegg-bin/show_pathway?mmu00760/mmu:103850%09red |
| Propanoate metabolism | KEGG PATHWAY | mmu00640 | 1 | 31 | 0.106952977 | 0.377879789 | Ldha | http://www.genome.jp/kegg-bin/show_pathway?mmu00640/mmu:16828%09red |
| Hippo signaling pathway | KEGG PATHWAY | mmu04390 | 2 | 156 | 0.10940036 | 0.377879789 | Tead1\|Bbc3 | http://www.genome.jp/kegg-bin/show_pathway?mmu04390/mmu:21676%09red/mmu:170770%09red |
| Prion diseases | KEGG PATHWAY | mmu05020 | 1 | 35 | 0.119923321 | 0.377879789 | Map2k2 | http://www.genome.jp/kegg-bin/show_pathway?mmu05020/mmu:26396%09red |
| Protein processing in endoplasmic reticulum | KEGG PATHWAY | mmu04141 | 2 | 169 | 0.124875786 | 0.377879789 | Dnaja1\|Hyou1 | http://www.genome.jp/kegg-bin/show_pathway?mmu04141/mmu:15502%09red/mmu:12282%09red |
| Influenza A | KEGG PATHWAY | mmu05164 | 2 | 171 | 0.127303761 | 0.377879789 | Map2k2\|Ikbke | http://www.genome.jp/kegg-bin/show_pathway?mmu05164/mmu:56489%09red/mmu:26396%09red |
| Cysteine and methionine metabolism | KEGG PATHWAY | mmu00270 | 1 | 38 | 0.129531837 | 0.377879789 | Ldha | http://www.genome.jp/kegg-bin/show_pathway?mmu00270/mmu:16828%09red |
| Bladder cancer | KEGG PATHWAY | mmu05219 | 1 | 39 | 0.132712167 | 0.377879789 | Map2k2 | http://www.genome.jp/kegg-bin/show_pathway?mmu05219/mmu:26396%09red |
| Fat digestion and absorption | KEGG PATHWAY | mmu04975 | 1 | 40 | 0.135881306 | 0.377879789 | Ppap2c | http://www.genome.jp/kegg-bin/show_pathway?mmu04975/mmu:50784%09red |
| Ether lipid metabolism | KEGG PATHWAY | mmu00565 | 1 | 41 | 0.139039293 | 0.377879789 | Ppap2c | http://www.genome.jp/kegg-bin/show_pathway?mmu00565/mmu:50784%09red |
| Huntington's disease | KEGG PATHWAY | mmu05016 | 2 | 182 | 0.140860224 | 0.377879789 | Atp5c1\|Bbc3 | http://www.genome.jp/kegg-bin/show_pathway?mmu05016/mmu:11949%09red/mmu:170770%09red |
| Basal transcription factors | KEGG PATHWAY | mmu03022 | 1 | 45 | 0.151560463 | 0.377879789 | Gtf2h2 | http://www.genome.jp/kegg-bin/show_pathway?mmu03022/mmu:23894%09red |
| Nucleotide excision repair | KEGG PATHWAY | mmu03420 | 1 | 45 | 0.151560463 | 0.377879789 | Gtf2h2 | http://www.genome.jp/kegg-bin/show_pathway?mmu03420/mmu:23894%09red |
| Sphingolipid metabolism | KEGG PATHWAY | mmu00600 | 1 | 48 | 0.160836073 | 0.377879789 | Ppap2c | http://www.genome.jp/kegg-bin/show_pathway?mmu00600/mmu:50784%09red |
| Herpes simplex infection | KEGG PATHWAY | mmu05168 | 2 | 204 | 0.168860691 | 0.377879789 | Hnrnpk\|Ikbke | http://www.genome.jp/kegg-bin/show_pathway?mmu05168/mmu:56489%09red/mmu:15387%09red |
| Endometrial cancer | KEGG PATHWAY | mmu05213 | 1 | 52 | 0.173051762 | 0.377879789 | Map2k2 | http://www.genome.jp/kegg-bin/show_pathway?mmu05213/mmu:26396%09red |
| Fanconi anemia pathway | KEGG PATHWAY | mmu03460 | 1 | 52 | 0.173051762 | 0.377879789 | 2310003H01Rik | http://www.genome.jp/kegg-bin/show_pathway?mmu03460/mmu:71885%09red |
| Non-small cell lung cancer | KEGG PATHWAY | mmu05223 | 1 | 55 | 0.182100966 | 0.377879789 | Map2k2 | http://www.genome.jp/kegg-bin/show_pathway?mmu05223/mmu:26396%09red |
| Regulation of actin cytoskeleton | KEGG PATHWAY | mmu04810 | 2 | 218 | 0.187172771 | 0.377879789 | Map2k2\|Baiap2 | http://www.genome.jp/kegg-bin/show_pathway?mmu04810/mmu:108100%09red/mmu:26396%09red |
| Acute myeloid leukemia | KEGG PATHWAY | mmu05221 | 1 | 57 | 0.188080712 | 0.377879789 | Map2k2 | http://www.genome.jp/kegg-bin/show_pathway?mmu05221/mmu:26396%09red |
| Cytosolic DNA-sensing pathway | KEGG PATHWAY | mmu04623 | 1 | 61 | 0.199914155 | 0.377879789 | Ikbke | http://www.genome.jp/kegg-bin/show_pathway?mmu04623/mmu:56489%09red |
| Inositol phosphate metabolism | KEGG PATHWAY | mmu00562 | 1 | 61 | 0.199914155 | 0.377879789 | Inpp4a | http://www.genome.jp/kegg-bin/show_pathway?mmu00562/mmu:269180%09red |
| Long-term depression | KEGG PATHWAY | mmu04730 | 1 | 61 | 0.199914155 | 0.377879789 | Map2k2 | http://www.genome.jp/kegg-bin/show_pathway?mmu04730/mmu:26396%09red |
| Viral carcinogenesis | KEGG PATHWAY | mmu05203 | 2 | 228 | 0.200435366 | 0.377879789 | Gtf2h2\|Hnrnpk | http://www.genome.jp/kegg-bin/show_pathway?mmu05203/mmu:15387%09red/mmu:23894%09red |
| Proteoglycans in cancer | KEGG PATHWAY | mmu05205 | 2 | 228 | 0.200435366 | 0.377879789 | Map2k2\|Cd44 | http://www.genome.jp/kegg-bin/show_pathway?mmu05205/mmu:26396%09red/mmu:12505%09red |
| Synaptic vesicle cycle | KEGG PATHWAY | mmu04721 | 1 | 62 | 0.202846464 | 0.377879789 | Tcirg1 | http://www.genome.jp/kegg-bin/show_pathway?mmu04721/mmu:27060%09red |
| VEGF signaling pathway | KEGG PATHWAY | mmu04370 | 1 | 62 | 0.202846464 | 0.377879789 | Map2k2 | http://www.genome.jp/kegg-bin/show_pathway?mmu04370/mmu:26396%09red |
| Glycolysis / Gluconeogenesis | KEGG PATHWAY | mmu00010 | 1 | 66 | 0.214472547 | 0.377879789 | Ldha | http://www.genome.jp/kegg-bin/show_pathway?mmu00010/mmu:16828%09red |
| Glioma | KEGG PATHWAY | mmu05214 | 1 | 66 | 0.214472547 | 0.377879789 | Map2k2 | http://www.genome.jp/kegg-bin/show_pathway?mmu05214/mmu:26396%09red |
| Long-term potentiation | KEGG PATHWAY | mmu04720 | 1 | 68 | 0.220224186 | 0.377879789 | Map2k2 | http://www.genome.jp/kegg-bin/show_pathway?mmu04720/mmu:26396%09red |
| Renal cell carcinoma | KEGG PATHWAY | mmu05211 | 1 | 68 | 0.220224186 | 0.377879789 | Map2k2 | http://www.genome.jp/kegg-bin/show_pathway?mmu05211/mmu:26396%09red |
| RIG-I-like receptor signaling pathway | KEGG PATHWAY | mmu04622 | 1 | 69 | 0.223084777 | 0.377879789 | Ikbke | http://www.genome.jp/kegg-bin/show_pathway?mmu04622/mmu:56489%09red |
| p53 signaling pathway | KEGG PATHWAY | mmu04115 | 1 | 69 | 0.223084777 | 0.377879789 | Bbc3 | http://www.genome.jp/kegg-bin/show_pathway?mmu04115/mmu:170770%09red |
| Fc epsilon RI signaling pathway | KEGG PATHWAY | mmu04664 | 1 | 71 | 0.228775672 | 0.377879789 | Map2k2 | http://www.genome.jp/kegg-bin/show_pathway?mmu04664/mmu:26396%09red |
| Melanoma | KEGG PATHWAY | mmu05218 | 1 | 72 | 0.231606046 | 0.377879789 | Map2k2 | http://www.genome.jp/kegg-bin/show_pathway?mmu05218/mmu:26396%09red |
| Prolactin signaling pathway | KEGG PATHWAY | mmu04917 | 1 | 74 | 0.237236814 | 0.377879789 | Map2k2 | http://www.genome.jp/kegg-bin/show_pathway?mmu04917/mmu:26396%09red |
| Chronic myeloid leukemia | KEGG PATHWAY | mmu05220 | 1 | 74 | 0.237236814 | 0.377879789 | Map2k2 | http://www.genome.jp/kegg-bin/show_pathway?mmu05220/mmu:26396%09red |
| Adherens junction | KEGG PATHWAY | mmu04520 | 1 | 75 | 0.240037277 | 0.377879789 | Baiap2 | http://www.genome.jp/kegg-bin/show_pathway?mmu04520/mmu:108100%09red |
| B cell receptor signaling pathway | KEGG PATHWAY | mmu04662 | 1 | 76 | 0.242827838 | 0.377879789 | Map2k2 | http://www.genome.jp/kegg-bin/show_pathway?mmu04662/mmu:26396%09red |
| Bacterial invasion of epithelial cells | KEGG PATHWAY | mmu05100 | 1 | 77 | 0.24560853 | 0.377879789 | Sept9 | http://www.genome.jp/kegg-bin/show_pathway?mmu05100/mmu:53860%09red |
| Peroxisome | KEGG PATHWAY | mmu04146 | 1 | 81 | 0.256633281 | 0.377879789 | Acot8 | http://www.genome.jp/kegg-bin/show_pathway?mmu04146/mmu:170789%09red |
| Phosphatidylinositol signaling system | KEGG PATHWAY | mmu04070 | 1 | 81 | 0.256633281 | 0.377879789 | Inpp4a | http://www.genome.jp/kegg-bin/show_pathway?mmu04070/mmu:269180%09red |
| Rheumatoid arthritis | KEGG PATHWAY | mmu05323 | 1 | 84 | 0.264799857 | 0.377879789 | Tcirg1 | http://www.genome.jp/kegg-bin/show_pathway?mmu05323/mmu:27060%09red |
| Hematopoietic cell lineage | KEGG PATHWAY | mmu04640 | 1 | 87 | 0.272880003 | 0.377879789 | Cd44 | http://www.genome.jp/kegg-bin/show_pathway?mmu04640/mmu:12505%09red |
| Retinol metabolism | KEGG PATHWAY | mmu00830 | 1 | 87 | 0.272880003 | 0.377879789 | Dhrs3 | http://www.genome.jp/kegg-bin/show_pathway?mmu00830/mmu:20148%09red |
| ECM-receptor interaction | KEGG PATHWAY | mmu04512 | 1 | 87 | 0.272880003 | 0.377879789 | Cd44 | http://www.genome.jp/kegg-bin/show_pathway?mmu04512/mmu:12505%09red |
| ErbB signaling pathway | KEGG PATHWAY | mmu04012 | 1 | 87 | 0.272880003 | 0.377879789 | Map2k2 | http://www.genome.jp/kegg-bin/show_pathway?mmu04012/mmu:26396%09red |
| Gap junction | KEGG PATHWAY | mmu04540 | 1 | 88 | 0.275554331 | 0.377879789 | Map2k2 | http://www.genome.jp/kegg-bin/show_pathway?mmu04540/mmu:26396%09red |
| Fc gamma R-mediated phagocytosis | KEGG PATHWAY | mmu04666 | 1 | 89 | 0.278219186 | 0.377879789 | Ppap2c | http://www.genome.jp/kegg-bin/show_pathway?mmu04666/mmu:50784%09red |
| Prostate cancer | KEGG PATHWAY | mmu05215 | 1 | 89 | 0.278219186 | 0.377879789 | Map2k2 | http://www.genome.jp/kegg-bin/show_pathway?mmu05215/mmu:26396%09red |
| GnRH signaling pathway | KEGG PATHWAY | mmu04912 | 1 | 89 | 0.278219186 | 0.377879789 | Map2k2 | http://www.genome.jp/kegg-bin/show_pathway?mmu04912/mmu:26396%09red |
| Estrogen signaling pathway | KEGG PATHWAY | mmu04915 | 1 | 99 | 0.304353759 | 0.404773957 | Map2k2 | http://www.genome.jp/kegg-bin/show_pathway?mmu04915/mmu:26396%09red |
| Melanogenesis | KEGG PATHWAY | mmu04916 | 1 | 100 | 0.306916516 | 0.404773957 | Map2k2 | http://www.genome.jp/kegg-bin/show_pathway?mmu04916/mmu:26396%09red |
| Pyrimidine metabolism | KEGG PATHWAY | mmu00240 | 1 | 102 | 0.312014784 | 0.405619219 | Nt5m | http://www.genome.jp/kegg-bin/show_pathway?mmu00240/mmu:103850%09red |
| T cell receptor signaling pathway | KEGG PATHWAY | mmu04660 | 1 | 108 | 0.327093829 | 0.419232936 | Map2k2 | http://www.genome.jp/kegg-bin/show_pathway?mmu04660/mmu:26396%09red |
| TNF signaling pathway | KEGG PATHWAY | mmu04668 | 1 | 110 | 0.332049074 | 0.419673135 | Dnm1l | http://www.genome.jp/kegg-bin/show_pathway?mmu04668/mmu:74006%09red |
| Natural killer cell mediated cytotoxicity | KEGG PATHWAY | mmu04650 | 1 | 122 | 0.361050767 | 0.443794812 | Map2k2 | http://www.genome.jp/kegg-bin/show_pathway?mmu04650/mmu:26396%09red |
| Neurotrophin signaling pathway | KEGG PATHWAY | mmu04722 | 1 | 123 | 0.363412063 | 0.443794812 | Map2k2 | http://www.genome.jp/kegg-bin/show_pathway?mmu04722/mmu:26396%09red |
| Lysosome | KEGG PATHWAY | mmu04142 | 1 | 124 | 0.365764955 | 0.443794812 | Tcirg1 | http://www.genome.jp/kegg-bin/show_pathway?mmu04142/mmu:27060%09red |
| Vascular smooth muscle contraction | KEGG PATHWAY | mmu04270 | 1 | 128 | 0.375093052 | 0.449124576 | Map2k2 | http://www.genome.jp/kegg-bin/show_pathway?mmu04270/mmu:26396%09red |
| Spliceosome | KEGG PATHWAY | mmu03040 | 1 | 132 | 0.384288965 | 0.454159686 | Hnrnpk | http://www.genome.jp/kegg-bin/show_pathway?mmu03040/mmu:15387%09red |
| Hepatitis C | KEGG PATHWAY | mmu05160 | 1 | 136 | 0.393354494 | 0.455691961 | Ikbke | http://www.genome.jp/kegg-bin/show_pathway?mmu05160/mmu:56489%09red |
| Parkinson's disease | KEGG PATHWAY | mmu05012 | 1 | 137 | 0.395600713 | 0.455691961 | Atp5c1 | http://www.genome.jp/kegg-bin/show_pathway?mmu05012/mmu:11949%09red |
| Insulin signaling pathway | KEGG PATHWAY | mmu04910 | 1 | 143 | 0.408910773 | 0.465136004 | Map2k2 | http://www.genome.jp/kegg-bin/show_pathway?mmu04910/mmu:26396%09red |
| Purine metabolism | KEGG PATHWAY | mmu00230 | 1 | 173 | 0.471337451 | 0.523215668 | Nt5m | http://www.genome.jp/kegg-bin/show_pathway?mmu00230/mmu:103850%09red |
| Alzheimer's disease | KEGG PATHWAY | mmu05010 | 1 | 176 | 0.477218686 | 0.523215668 | Atp5c1 | http://www.genome.jp/kegg-bin/show_pathway?mmu05010/mmu:11949%09red |
| Phagosome | KEGG PATHWAY | mmu04145 | 1 | 176 | 0.477218686 | 0.523215668 | Tcirg1 | http://www.genome.jp/kegg-bin/show_pathway?mmu04145/mmu:27060%09red |
| Tuberculosis | KEGG PATHWAY | mmu05152 | 1 | 179 | 0.483036891 | 0.523289966 | Tcirg1 | http://www.genome.jp/kegg-bin/show_pathway?mmu05152/mmu:27060%09red |
| Focal adhesion | KEGG PATHWAY | mmu04510 | 1 | 206 | 0.532669507 | 0.570269708 | Tln1 | http://www.genome.jp/kegg-bin/show_pathway?mmu04510/mmu:21894%09red |
| Epstein-Barr virus infection | KEGG PATHWAY | mmu05169 | 1 | 211 | 0.541342571 | 0.572815976 | Cd44 | http://www.genome.jp/kegg-bin/show_pathway?mmu05169/mmu:12505%09red |
| Endocytosis | KEGG PATHWAY | mmu04144 | 1 | 220 | 0.556565012 | 0.582154208 | Git2 | http://www.genome.jp/kegg-bin/show_pathway?mmu04144/mmu:26431%09red |
| MAPK signaling pathway | KEGG PATHWAY | mmu04010 | 1 | 258 | 0.615642115 | 0.636629914 | Map2k2 | http://www.genome.jp/kegg-bin/show_pathway?mmu04010/mmu:26396%09red |
| HTLV-I infection | KEGG PATHWAY | mmu05166 | 1 | 280 | 0.646299496 | 0.66082308 | Tln1 | http://www.genome.jp/kegg-bin/show_pathway?mmu05166/mmu:21894%09red |
| Pathways in cancer | KEGG PATHWAY | mmu05200 | 1 | 324 | 0.700704418 | 0.708490023 | Map2k2 | http://www.genome.jp/kegg-bin/show_pathway?mmu05200/mmu:26396%09red |
| PI3K-Akt signaling pathway | KEGG PATHWAY | mmu04151 | 1 | 352 | 0.731028222 | 0.731028222 | Map2k2 | http://www.genome.jp/kegg-bin/show_pathway?mmu04151/mmu:26396%09red |
