# Supplementary material for: CPEB3 regulates neuron-specific alternative splicing and involves neurogenesis gene expression
Source: Aging (Albany NY). 2020 Dec 9;13(2):2330–47. doi: 10.18632/aging.202259 (PMC7880327; doi:10.18632/aging.202259)
Supplement: Additional File 1 [file aging-13-202259-s007.docx]

Additional file 1. Primers sets, related to the experimental procedures. qRT-PCR primers for gene expression qualification.

| **Gene** | **Primer** | **Sequence (5'-3')** | **Related Figures** |
| --- | --- | --- | --- |
| GAPDH | Forward | CGGAGTCAACGGATTTGGTCGTAT | Figure 1.A |
|  | Reverse | AGCCTTCTCCATGGTGGTGAAGAC |  |
| CPEB3 | Forward | GATACGGACCCAGAGCTGAA | Figure 1.A |
|  | Reverse | CCTGGCACTCATCACACATC |  |
